# Assessing the Incremental Value of Environment-Aware Bioelectrical Impedance Sensing for Machine Learning-Based Anemia Risk Monitoring in Goats

**DOI:** 10.3390/ani16213441

**Published:** 2026-10-31

**Authors:** Ramya Kota, Shreya Neelagiri, Goutham Kumar Isai, Davia Brown, Jayanta Banik, Andrea Villa Rubio, Phaneendra Batchu, David Shapiro-Ilan, Cristina Pisani, Aftab Siddique, Ajit K. Mahapatra, Sudhanshu S. Panda, Thomas H. Terrill

**Affiliations:** 1 Department of Agricultural Sciences, Fort Valley State University, Fort Valley, GA 31030, USA; rkota@wildcat.fvsu.edu (R.K.); sneelagi@wildcat.fvsu.edu (S.N.); gisai@wildcat.fvsu.edu (G.K.I.); dbrow212@wildcat.fvsu.edu (D.B.); jayanta.banik@fvsu.edu (J.B.); arubiovi@wildcat.fvsu.edu (A.V.R.); phaneendra.batchu@fvsu.edu (P.B.); mahapatraa@fvsu.edu (A.K.M.); terrillt@fvsu.edu (T.H.T.); 2 USDA-ARS, Fruit and Tree Nut Research Station, Byron, GA 31008, USA; david.shapiro@usda.gov (D.S.-I.); cristina.pisani@usda.gov (C.P.); 3 Institute for Environmental Spatial Analysis, University of North Georgia, 3820 Mundy Mill Road, Oakwood, GA 30566, USA; sudhanshu.panda@ung.edu

**Keywords:** anemia, bioelectrical impedance analysis, FAMACHA, monitoring, machine learning, precision livestock

## Abstract

Bioelectrical impedance analysis (BIA) has been widely evaluated in livestock for body composition and physiological assessment, but its incremental value for anemia-risk monitoring in small ruminants remains unclear. This study evaluated BIA as part of an environmentally aware multimodal machine-learning framework for anemia-related monitoring in goats. Data from 81 Spanish male goats were restructured into animal-by-date observations and integrated with objective FAMACHA© classification, blood packed cell volume, gastrointestinal nematode fecal egg count, weather variables, temperature–humidity index, and raster-derived environmental covariates. Objective FAMACHA© was derived from packed cell volume and used as the primary classification endpoint. Binary, three-class, and four-class classification tasks were evaluated using animal-grouped cross-validation to reduce leakage from repeated observations. Binary low-risk versus high-risk classification was the most reliable task, whereas multiclass prediction showed limited resolution among adjacent FAMACHA© categories. A formal ablation analysis showed that BIA-only features achieved a ROC-AUC of 0.672, compared with 0.838 for fecal egg count plus weather/THI and 0.824 for the complete multimodal model. The full model did not significantly outperform the fecal egg count plus weather/THI model (ΔAUC = −0.013; 95% CI: −0.124 to 0.093; *p* = 0.844). A supplementary matched feature-block analysis likewise did not demonstrate a statistically reliable incremental improvement from adding BIA. Threshold analysis showed that a screening-oriented threshold detected 83.3% of high-risk observations with 71.3% specificity. These findings indicate that parasitological and environmental predictors provided the strongest discriminatory information, whereas the independent incremental predictive benefit of BIA remained unestablished. BIA should therefore be regarded as a potential supplementary physiological sensing modality rather than a validated primary predictor or replacement for established anemia-assessment methods.

## Simple Summary

Anemia from blood-feeding parasites is a serious health and productivity issue for goats. To find affected animals, people often check the color of the lower eyelid, test blood for red blood cell levels, or count parasite eggs in feces. However, these methods can need special training, animal handling, or lab work. In this study, we tested if bioelectrical impedance analysis—a quick way to see how body tissues react to a small electrical current—could help spot goats at higher risk of anemia. We used data from 81 goats, including blood tests, parasite egg counts, eye-color scores, weather, and environmental details. Electrical measurements alone did not predict anemia well, but when we combined them with other information, we could better identify animals at higher risk. In an exploratory evaluation, the screening threshold identified 83.3% of higher-risk analytical observations and correctly classified 71.3% of lower-risk observations. These results suggest that bioelectrical impedance should not replace current anemia tests, but it could be a helpful extra tool in future automated systems for monitoring livestock health.

## 1. Introduction

Goats are increasingly important to global food systems because they contribute to household nutrition, income generation, ecological adaptability, and resilience in resource-constrained and climate-stressed production environments [1]. At the same time, small-ruminant systems face growing pressure from climate variability, pasture instability, health constraints, and the need for more sustainable management, especially where parasitism remains a major cause of productivity loss [1,2,3,4]. In this context, improving the detection and management of anemia in goats is not only a veterinary issue but also a practical challenge in sustainable production, animal welfare, and precision livestock farming.

Among the major health constraints in goats, infection with the gastrointestinal nematode *Haemonchus contortus* remains one of the most consequential because of its blood-feeding habit, high fecundity, rapid transmission dynamics, and ability to cause severe anemia, reduced growth, reproductive losses, and mortality under field conditions [5,6,7,8,9,10]. The challenge is compounded by widespread and often multidrug anthelmintic resistance, which has shifted parasite control away from blanket deworming toward more selective and evidence-based treatment strategies [3,11,12,13,14,15]. This transition has increased the need for practical monitoring tools and decision-support systems that can identify animals most likely to benefit from treatment while helping preserve refugia and reduce resistance selection pressure.

The FAMACHA© system has become one of the most influential targeted selective treatment tools because it provides a rapid, low-cost, on-farm estimate of anemia severity based on ocular mucous membrane color [5,16,17,18]. It has been validated in sheep and goats and remains widely used because it can reduce necessary treatment while helping identify clinically affected animals [5,6,9]. However, FAMACHA© is not without limitations. Its performance can vary with species, parasite challenge, operator skill, regional conditions, and the biological relationship among anemia, fecal egg counts, and production traits [9,15,19,20,21,22]. These limitations have created a practical need for complementary approaches that can strengthen field decision-making with more objective physiological, parasitological, and sensor-derived information.

Blood packed cell volume (PCV) remains one of the most direct and clinically interpretable hematologic indicators of anemia in small ruminants and therefore provides a stronger physiological reference than field FAMACHA© alone when evaluating diagnostic and predictive methods [5,6]. Yet, PCV measurement is invasive, labor-intensive, and less convenient for repeated or large-scale field monitoring. This gap has encouraged interest in non-invasive or minimally invasive proxies that can complement conventional clinical scoring. In practice, selective treatment decisions are strongest when they are supported by objective biological information rather than solely by visual assessment, especially under conditions of parasite resistance, mixed parasite pressure, or variable operator experience [11,23,24].

Bioelectrical impedance analysis (BIA) offers one such sensing framework. In its simplest form, BIA quantifies the opposition of biological tissues to an applied electrical current through resistance and reactance, from which impedance magnitude and phase angle can be derived [25,26]. These variables have long been studied in human medicine and nutrition because they reflect fluid distribution, tissue composition, and membrane-related electrical properties, and phase angle is often interpreted as a marker associated with cell integrity and hydration state [25,26]. Although BIA is not a direct measure of anemia, its sensitivity to body fluids, tissue conductivity, and physiological status raises an important question: can anemia-related changes in goats produce detectable electrical signatures that are useful for smart livestock monitoring? This remains uncertain because impedance signals may be influenced by hydration, body condition, parasite burden, and other contextual factors rather than by anemia-specific physiology alone.

Interest in BIA use in animal systems has grown, but most applications have historically focused on carcass composition, nutrient retention, and body composition estimation rather than clinical hematology or parasite-linked health monitoring [27,28,29]. This creates an important research gap. If BIA-derived variables can contribute to classification of anemia-related risk states or prediction of PCV in goats, then BIA may serve as a useful non-invasive sensing input for precision livestock health systems. At the same time, because impedance measurements are influenced by multiple biological and environmental factors, their value is likely to be greatest when interpreted within a multimodal framework rather than as an isolated biomarker.

These questions fit naturally within the broader development of precision livestock farming, where sensors, structured data streams, and machine learning are increasingly used to improve animal monitoring, treatment timing, and management efficiency [4,30,31,32,33,34]. Precision livestock systems are most useful when they move beyond simple signal collection to provide interpretable outputs and field-relevant decision support under real production variability [35,36,37]. In this sense, combining BIA-derived measurements with supervised learning and contextual data is not merely a technical exercise. It is a test of whether a physiology-linked electrical signal can contribute to practical, data-driven monitoring of anemia-related status in goats.

Our recent work showed that BIA combined with machine learning could differentiate goats exposed to hemonchosis, suggesting that impedance-derived information may have value in parasite-related monitoring [38]. However, the academic and practical value of BIA for anemia-risk monitoring depends not only on whether impedance variables signal parasitic infection, but also on whether they add measurable predictive value beyond simpler biological and environmental predictors. The innovation of the present study is therefore not the proposal of a new machine-learning algorithm, but the development and validation of an environment-aware multimodal sensing framework for goat anemia-risk monitoring. Specifically, the study integrates PCV-derived objective FAMACHA© classification, BIA-derived electrical features, fecal parasite burden, weather variables, thermal-humidity index (THI), and raster-derived environmental covariates within an animal-grouped analytical structure. We hypothesized that (1) incorporating BIA-derived features into a conventional predictor set comprising fecal egg count and weather/THI variables would improve anemia-risk discrimination and (2) multimodal integration of physiological, parasitological, and environmental features would provide stronger predictive performance than BIA-only models. These hypotheses were evaluated using standardized feature-block ablation and supplementary matched-model comparisons to distinguish the independent contribution of BIA from the broader effects of multimodal integration.

## 2. Materials and Methods

### 2.1. Study Design and Analytical Framework

**Figure 1:**
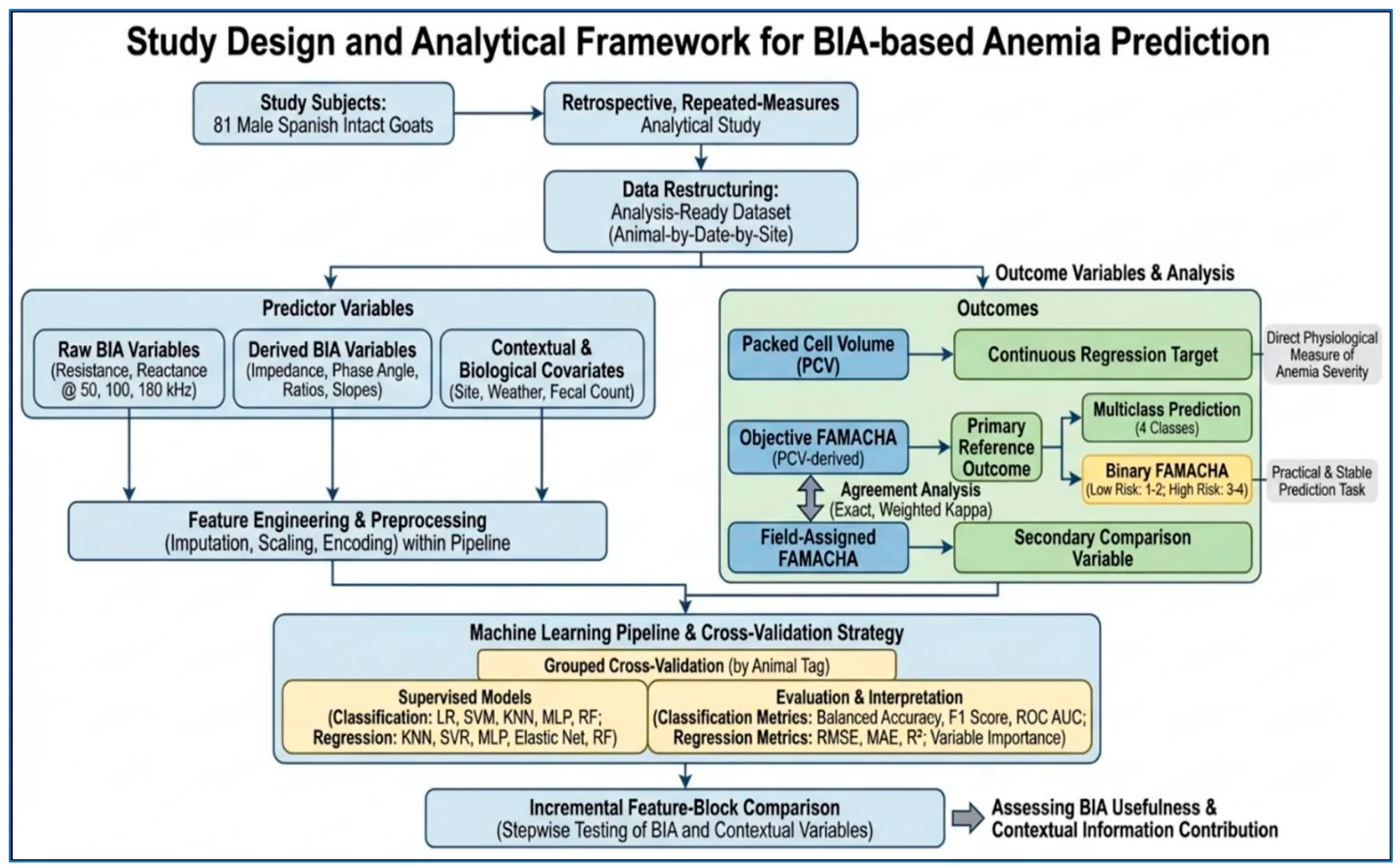
Conceptual overview of the study design and analytical workflow used to evaluate bioelectrical impedance analysis, contextual variables, and machine learning for anemia-related monitoring in goats.

This study was designed as a repeated-measures analytical investigation to evaluate whether bioelectrical impedance analysis (BIA) variables could support classification of objective FAMACHA© status and prediction of packed cell volume (PCV) in goats. A total of 81 Spanish intact male goats (24 months old; 36–50 kg) were included. The analytical framework combined agreement analysis, repeated-measures statistical modeling, and supervised machine-learning methods to evaluate the role of BIA within a multimodal monitoring system. PCV-derived objective FAMACHA© was treated as the primary reference outcome, whereas field-assigned FAMACHA© was retained as a secondary comparison variable. This structure is consistent with the biological basis of FAMACHA©, which was originally developed as a field proxy for anemia severity associated with *Haemonchus contortus* infection and validated against hematologic indicators such as PCV [5,6]. The overall study design and analytical workflow are summarized in Figure 1.

### 2.2. Data Restructuring and Analysis-Ready Dataset

The original dataset consisted of scan-level BIA measurements collected repeatedly from goats across sampling occasions. Because multiple BIA readings were acquired from the same goat during a given sampling event, the raw records were restructured into an analysis-ready dataset in which repeated scans from the same goat on the same date and site were summarized into a single animal-by-date-by-site observation. This approach preserved animal-level grouping while reducing pseudo replication and aligning the observation structure with PCV, objective FAMACHA©, fecal egg count, and contextual covariates. Summary descriptors derived from the repeated scans included central tendency, variability, scan-count, and derived impedance features. BIA measurements were organized by animal identification, sampling date, anatomical measurement site, and measurement frequency (50, 100, and 180 kHz). Repeated readings within each animal-by-date-by-site combination were summarized using the mean and standard deviation of resistance and reactance, while the number of recorded scans at each frequency was retained as a separate variable. In the archived analysis-ready dataset, the median number of recorded scans per available frequency and sampling record was 12 at 50 kHz (range: 1–27), 12 at 100 kHz (range: 2–26), and 11 at 180 kHz (range: 1–31). Frequency availability varied across sampling records. These values represent retained scan counts rather than measurements independently verified against a documented signal-quality acceptance standard. The available analytical records did not provide sufficient information to retrospectively verify the consistency of instrument calibration, electrode contact, skin preparation, animal posture, or hydration status across all measurements.

The resulting analysis-ready dataset comprised 183 animal-by-date-by-site observations from 81 goats. Two observations from two goats lacked the PCV-derived objective FAMACHA reference classification and were excluded from classification analyses, leaving 181 labeled observations from 79 goats. Individual goats contributed between one and five labeled observations, including measurements obtained from different anatomical sites on the same date and repeated measurements across collection dates. Consequently, the summarized observation count should not be interpreted as the number of statistically independent animals or independent longitudinal sampling events. Animal identity was retained throughout the analysis to account for repeated measurements and to prevent information leakage between training and testing partitions.

### 2.3. Outcome Variables

#### 2.3.1. Objective and Field FAMACHA©

Unlike our previous study, which evaluated BIA as a tool for classifying goats according to hemonchosis exposure status, the present study used objective FAMACHA© as the primary classification endpoint. Objective FAMACHA©, derived from PCV, served as the reference label for predictive modeling, whereas field FAMACHA© was retained for agreement analysis. In addition to multiclass prediction, a binary FAMACHA© outcome was defined to separate low-risk animals from high-risk animals, with classes 1 (PCV > 28%) and 2 (PCV between 22–28%) grouped as low risk and classes 3 (PCV between 17–21%) and 4 (PCV between 12–17%), and PCV < 12% for class 5 grouped as high risk. This binary structure was included because the most practical and stable prediction task in the current dataset was discrimination between clinically lower-risk and higher-risk animals rather than fine separation across all four classes. This is also consistent with the management logic of FAMACHA©, which is most actionable when used to distinguish animals requiring treatment attention from those that do not [5,38].

#### 2.3.2. Packed Cell Volume as a Regression Target

Packed Cell Volume was used as the continuous regression target because it provides a direct physiological measure of anemia severity. Including PCV as a regression outcome allowed the analysis to move beyond categorical classification and test whether BIA-derived signals tracked underlying hematologic variation. This is consistent with the original validation rationale of FAMACHA©, where eye score functions as a practical field indicator of anemia rather than a replacement for direct blood-based measurement [5].

### 2.4. Predictor Variables and Feature Engineering

#### 2.4.1. Raw BIA Variables

Predictor variables were first organized into structured feature blocks. The first block consisted of raw BIA variables, including resistance and reactance measured at 50, 100, and 180 kHz, together with standard deviation and scan-count variables where available.

#### 2.4.2. Derived BIA Variables

A second feature block expanded the raw BIA measurements with derived electrical variables generated from the impedance signals. These included impedance magnitude, phase angle, cross-frequency ratios, differences between frequencies, and slope-based descriptors across the measured frequencies. This feature engineering approach is consistent with established BIA methodology because resistance, reactance, impedance magnitude, and phase angle represent core descriptors of tissue conductivity, membrane capacitance, hydration status, and body-composition-related electrical behavior [25,26]. Impedance magnitude was calculated as *Z* = √*R*^2^ + *X*^2^, where *R*c is resistance and *X*c is reactance. Phase angle was calculated as the arctangent of reactance divided by resistance and expressed in degrees. Additional engineered variables included lower-frequency to higher-frequency ratios, cross-frequency deltas, and slope terms designed to capture how electrical responses changed across frequencies. These transformations were included because derived impedance features may contain biologically relevant information not captured by raw single-frequency measurements alone [25,26].

#### 2.4.3. Site, Weather, and Biological Covariates

Beyond BIA-derived features, the analytical workflow incorporated site, weather, and biological covariates into incremental feature blocks. Site was treated as a categorical contextual variable. Weather covariates included temperature, rainfall, wind speed, relative humidity, dew point, soil-related variables, and wind direction. Wind direction was transformed into angular, sine, and cosine components to preserve circular information in a numerically interpretable form. Additional biological variables, particularly fecal egg count, were included in later model blocks to test whether contextual and parasitological information improved predictive performance beyond BIA alone.

#### 2.4.4. Raster-Derived Environmental Covariates and THI

**Figure 2:**
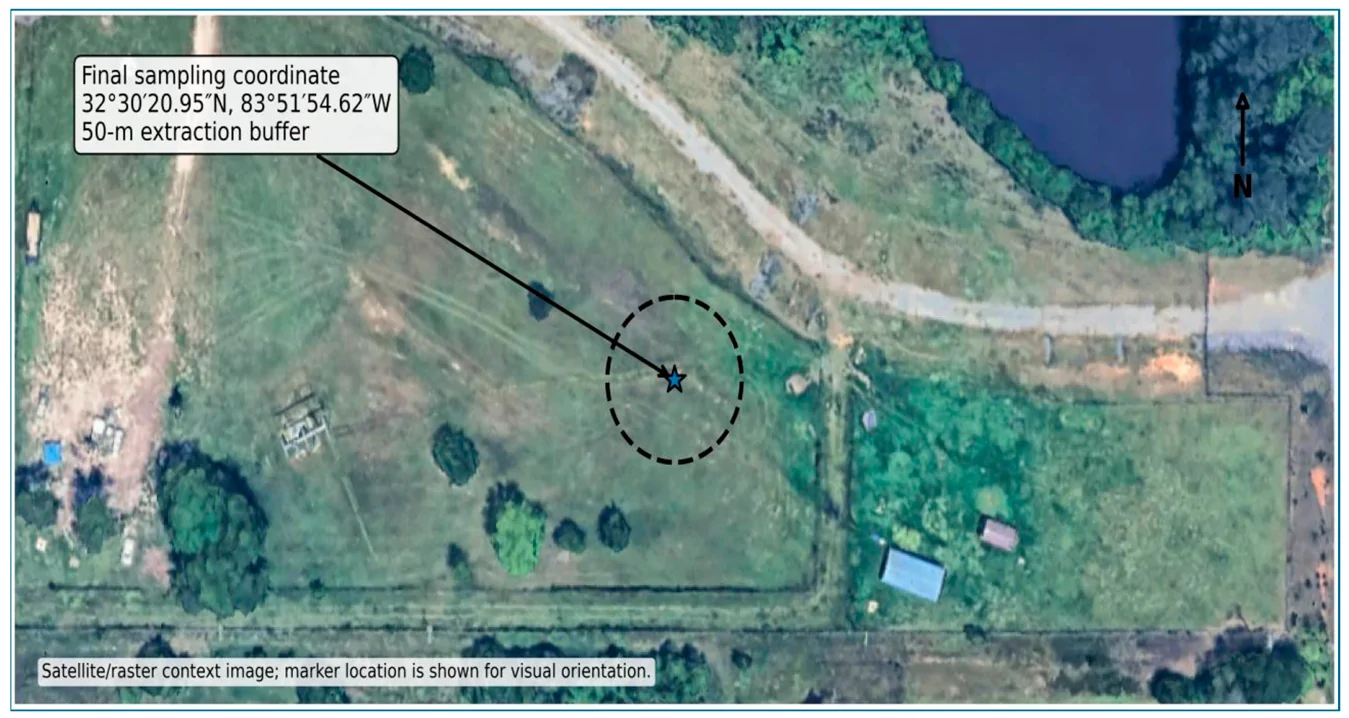
Site context image showing the final goat sampling coordinate and raster-extraction area. The final sampling coordinate was 32°30′20.95″ N, 83°51′54.62″ W. A 50 m local extraction buffer was used to derive raster-based environmental covariates, including NDVI, land surface temperature, elevation, slope, and land-cover context. The image provides visual site context for the pasture/raster environment used in the multimodal anemia-monitoring framework.

Temperature–humidity index (THI) was calculated from daily air temperature and relative humidity to provide an additional thermal-stress indicator for each sampling date. The THI was calculated as: THI = (1.8 × temperature (°C) + 32) − [(0.55 − 0.0055 × relative humidity %) × (1.8 × temperature (°C) − 26)]. Raster-derived environmental covariates were also extracted for the final sampling coordinate, 32°30′20.95″ N, 83°51′54.62″ W, using a 50 m buffer around the sampling location. Date-specific normalized difference vegetation index (NDVI) was extracted from Sentinel-2 surface reflectance using a ±7-day window around each sampling date. Land surface temperature (LST) was extracted from Landsat 8/9 surface temperature products using a ±16-day window. Environmental covariates were temporally aligned with animal sampling dates using the specified observation windows, while raster-derived values represented the common sampling location and its 50 m buffer rather than individual animal-level exposure. Consequently, these measurements provided date-specific environmental context but did not capture spatial variation in exposure among individual goats. Static geographical variables, including elevation, slope, and land-cover classification, were extracted from geospatial raster datasets exclusively to characterize the study location. Because all observations originated from a single geographical site, these variables did not provide independent spatial variation and were excluded from all machine-learning predictor sets, including the raster-derived and full multimodal feature blocks. In contrast, date-specific NDVI and land surface temperature (LST) were evaluated as temporally varying environmental covariates. The final sampling location and 50 m raster extraction buffer are shown in Figure 2.

### 2.5. Data Preprocessing

Data preprocessing was performed within the machine-learning workflow using scikit-learn components. Numerical predictors were imputed using the median and, where appropriate, standardized by z-score transformation depending on model family. Categorical predictors were imputed using the most frequent category and one-hot encoded before model fitting. This approach ensured that preprocessing remained embedded within the same resampling framework as model training and testing, thereby reducing the risk of data leakage. The analysis was implemented in Python (Version 3.10) using pandas, NumPy, SciPy, stats models, and scikit-learn, which are widely used for reproducible statistical computing and machine-learning workflows.

### 2.6. Exploratory and Inferential Statistical Analysis

#### 2.6.1. Exploratory Data Analysis

Exploratory data analysis included dataset summary, missingness assessment, class distribution, descriptive statistics by objective FAMACHA© class, and correlation analysis between BIA variables and PCV. Descriptive summaries included count, mean, standard deviation, median, quartiles, and interquartile range. Because the dataset contained repeated measurements from the same animals and uneven representation across FAMACHA© classes, descriptive comparisons were interpreted alongside repeated-measures inferential models rather than as standalone evidence of class separation.

#### 2.6.2. Repeated-Measures Mixed-Model Analysis

To evaluate class-level differences in BIA variables while accounting for repeated observations within animals, each BIA variable was analyzed using a repeated-measures mixed-model framework. Objective FAMACHA© class was treated as a fixed effect, and animal identity was included as a random effect to account for within-animal correlation. This framework was used to estimate overall class effects, least-squares means, and pairwise differences among FAMACHA© classes. For manuscript-ready mean separation, post hoc group comparisons were generated using Tukey-style multiple comparison procedures on class-level contrasts. Mean ± standard deviation summaries with superscript groupings were also prepared to facilitate table presentation. Because FAMACHA© classes are ordinal and class sizes were unequal, this mixed-model framework provided a more appropriate inferential approach than simpler one-way group comparisons.

#### 2.6.3. Frequency-Wise Comparison of BIA Families

To determine whether frequency-dependent behavior differed systematically across objective FAMACHA© classes, additional repeated-measures mixed models were fit for grouped BIA families spanning 50, 100, and 180 kHz. These families included resistance mean, reactance mean, impedance magnitude, phase angle, resistance standard deviation, reactance standard deviation, and scan count. In these models, FAMACHA© class, frequency, and their interaction were treated as fixed effects, and animal identity was included as a random effect. This allowed evaluation of overall class effects, frequency effects, and FAMACHA© × frequency interaction effects.

#### 2.6.4. Correlation Analysis

Spearman rank correlations were calculated to explore monotonic associations between BIA-derived variables and packed cell volume (PCV). Preliminary Shapiro–Wilk and Levene tests, together with conventional ANOVA and Kruskal–Wallis comparisons, were retained as exploratory diagnostics (Supplementary Table S1) rather than used to determine the primary inferential framework, because these conventional tests do not account for repeated observations within animals. The primary inferential analyses were therefore based on mixed-effects models incorporating animal identity as a random effect. Spearman correlations were interpreted as exploratory associations, and their *p*-values were adjusted across 57 comparisons using the Benjamini–Hochberg false discovery rate (FDR) procedure. For the two strongest nominal associations, 95% confidence intervals for Spearman’s rho were additionally estimated using 6000 animal-clustered bootstrap replicates.

### 2.7. Agreement Analysis Between Objective and Field FAMACHA©

Agreement between objective FAMACHA© and field FAMACHA© was evaluated using exact agreement and quadratic weighted kappa. Exact agreement quantified the proportion of observations for which the two labels matched directly. Quadratic weighted kappa was used because disagreement between adjacent FAMACHA© classes is less severe than disagreement between more distant classes, making weighted agreement more appropriate for an ordinal clinical scoring system.

### 2.8. Machine-Learning Models

#### 2.8.1. Classification Models

For supervised classification, five model families were evaluated: logistic regression, support vector machine, k-nearest neighbors, multilayer perceptron, and random forest. These models were chosen to compare linear, kernel-based, instance-based, neural-network, and ensemble-learning approaches within the same feature-block structure.

#### 2.8.2. Regression Models

For continuous PCV regression, the workflow evaluated k-nearest neighbors’ regression, support vector regression, multilayer perceptron regression, Elastic Net, and random forest regression. These models were selected to assess whether BIA-derived and contextual variables could predict hematologic variation using a range of linear and nonlinear strategies.

### 2.9. Cross-Validation Strategy

All machine-learning models were fit using grouped cross-validation by animal tag, implemented through GroupKFold, so that observations from the same animal were not split between training and testing folds. This was a critical design feature because repeated observations from the same animal are not independent, and leakage across folds would inflate performance estimates. To characterize the stability of the animal-grouped evaluation, the distribution of outcome classes and unique animals across five held-out folds was additionally examined. The folds were reconstructed using GroupKFold with animal tag as the grouping variable. The resulting class distributions were summarized descriptively, with particular attention to the number of independent high-risk animals and representation of the severe-anemia category.

Although animal-grouped cross-validation reduced the risk of information leakage between training and testing subsets, model evaluation was restricted to the original study population. No independent external dataset from different goat breeds, farms, or management systems was available for validation. Consequently, the reported predictive performance reflects internal cross-validation rather than external generalizability across livestock production environments.

### 2.10. Model Evaluation and Interpretation

Classification performance was quantified using balanced accuracy, macro F1 score, weighted F1 score, area under the receiver operating characteristic curve, average precision, and Cohen’s kappa. Balanced accuracy and macro F1 were emphasized because class imbalance was present and overall accuracy alone would be misleading. Regression performance was quantified using root mean squared error, mean absolute error, and coefficient of determination (*R*^2^). Permutation importance was applied to the best-performing binary classifier and the best-performing regression model to identify the variables contributing most strongly to predictive performance. This generated an interpretable ranking of BIA and non-BIA variables associated with classification of anemia-related risk and prediction of PCV.

In addition to the primary model-family comparison, a standardized ablation analysis was conducted for the binary low-risk versus high-risk objective FAMACHA© task. This analysis used balanced logistic regression across all feature blocks so that differences in performance reflected feature-block composition rather than changes in model architecture. Six feature blocks were evaluated: BIA only, BIA plus fecal count, BIA plus weather/THI, BIA plus raster-derived environmental covariates, fecal egg count plus weather/THI, and the full multimodal feature block. Blood PCV was excluded from all predictive feature blocks because it was used to derive the objective FAMACHA© reference endpoint. Field FAMACHA© was also excluded from model inputs because it represented a clinical comparator rather than an independent sensor-derived predictor.

Incremental model improvement was evaluated using paired bootstrap comparisons of out-of-fold predicted probabilities. Differences in ROC-AUC were calculated between the full multimodal model and each reduced feature block, with 95% confidence intervals and two-sided *p*-values estimated from the bootstrap distribution.

To further investigate the independent incremental predictive contribution of BIA, a supplementary matched feature-block sensitivity analysis was conducted using the archived analysis-ready dataset. Of the 183 summarized observations, 181 observations from 79 goats had available objective FAMACHA classifications and were included in binary low-risk (classes 1–2) versus high-risk (classes 3–4) classification. Two balanced logistic regression models were compared: a conventional baseline model comprising 17 fecal egg count and weather/THI predictors and an augmented model containing the same predictors plus 42 BIA-derived features. Both models were evaluated using identical five-fold animal-grouped cross-validation splits, with imputation, scaling, and categorical encoding performed within the training folds. Incremental discrimination was quantified as the difference in out-of-fold ROC-AUC between the BIA-augmented and conventional models. Paired bootstrap resampling at the animal level (6000 replicates) was used to estimate 95% confidence intervals while accounting for repeated observations within goats. Additional sensitivity analyses excluded BIA scan-count features, examined alternative animal-grouped cross-validation splits, and restricted the comparison to observations with measured fecal egg counts. Because this supplementary analysis was reconstructed from an archived analysis-ready dataset, its results were evaluated separately from the original six-block ablation analysis rather than treated as an exact replication of the original modeling pipeline.

To assess practical decision-support value, threshold analysis was performed using out-of-fold predicted probabilities from the full multimodal model. The Youden J threshold and a sensitivity-oriented threshold of 0.13 were examined to evaluate the trade-off between identifying high-risk animals and maintaining specificity. Data-dependent preprocessing was incorporated within the model-training workflow, with imputation, scaling, and categorical encoding fitted within training folds in the supplementary matched-model analysis. Candidate model families and feature blocks were evaluated using animal-grouped cross-validation; however, the available original analysis records do not establish that model selection or hyperparameter optimization was performed within an independent nested validation procedure. Similarly, independent selection or prespecification of the 0.13 threshold could not be verified. Consequently, threshold-specific performance estimates should be interpreted as exploratory because selection-related optimism cannot be excluded.

## 3. Results

### 3.1. Dataset Structure and Agreement Between Objective and Field FAMACHA©

The original dataset contained 36,215 raw scan-level BIA measurement records collected across frequencies, animals, anatomical measurement sites, and sampling occasions. Following aggregation, the analysis-ready dataset comprised 183 animal-by-date-by-site observations from 81 goats. Two observations lacked the PCV-derived objective FAMACHA reference classification and were excluded from classification analyses, resulting in 181 labeled observations from 79 goats. The distribution of labeled observations per goat was uneven: 33 goats contributed one observation, 15 contributed two, 13 contributed three, 11 contributed four, and seven contributed five observations (median = 2; range = 1–5). However, not all repeated observations represented independent sampling dates. Specifically, 48 goats were represented on one collection date, 23 on two dates, and eight on three dates. The objective FAMACHA outcome was strongly imbalanced. Class 1 comprised 85 observations from 40 unique goats, class 2 comprised 72 observations from 41 goats, class 3 comprised 21 observations from 19 goats, and class 4 comprised only three observations from three goats. Animal counts across classes were not mutually exclusive because individual goats could contribute observations to different categories over time. Following binary grouping, the low-risk category (classes 1–2) contained 157 observations, whereas the high-risk category (classes 3–4) contained only 24 observations from 21 unique goats. Among the 31 goats evaluated on multiple sampling dates, 11 exhibited changes between low- and high-risk classifications. Thus, although the analytical dataset contained 181 labeled observations, the effective amount of independent animal-level information was substantially smaller, particularly for higher-risk classifications.

In the animal-grouped five-fold cross-validation audit, the number of high-risk observations per held-out fold ranged from three to six, representing three to five unique high-risk goats. Two of the five reconstructed folds contained no class 4 observations. These findings highlight the limited representation of higher-risk animals and the potential instability of class-specific performance estimates. Detailed animal-level distributions and cross-validation fold characteristics are presented in Supplementary Tables S9 and S10, respectively.

Agreement analysis between objective and field-based FAMACHA© showed strong concordance. Across 690 paired records in the underlying dataset, exact agreement between the two systems was 94.49%, and the quadratic weighted kappa was 0.934. Most disagreements occurred between adjacent categories rather than distant classes, indicating that field scoring remained broadly consistent with the PCV-derived objective classification. These findings support the continued practical value of field FAMACHA© while also justifying the use of objective FAMACHA© as the reference endpoint for model development.

### 3.2. Descriptive Trends Across Objective FAMACHA© Classes

Descriptive summaries showed the expected physiological ordering across objective FAMACHA© classes. Mean PCV declined progressively from 31.23% in class 1 to 25.55% in class 2, 20.43% in class 3, and 15.83% in class 4, confirming that the objective categorization preserved the biological gradient of anemia severity. Fecal count increased in the opposite direction, rising from 268.47 in class 1 to 758.57 in class 2, 2187.50 in class 3, and 4800.60 in class 4. Although the estimate for class 4 should be interpreted cautiously because of the small number of observations, the overall pattern remained biologically coherent and consistent with worsening parasite-associated anemia.

Several BIA-derived variables also showed descriptive variation across classes. At 180 kHz, mean phase angle increased from 29.90° in class 1 to 36.08° in class 2, 33.28° in class 3, and 38.24° in class 4. Mean reactance at 180 kHz was 540.51, 723.05, 532.40, and 710.54 across classes 1 through 4, respectively, whereas impedance magnitude at 180 kHz was 823.05, 1083.44, 972.93, and 1046.07. These descriptive trends suggested that higher-frequency impedance features might be responsive to class differences, but the direction of change was not fully monotonic and substantial within-class variability was evident.

### 3.3. Repeated-Measures Mixed-Model Comparison of BIA Variables Across FAMACHA© Classes

When class-level differences were evaluated using repeated-measures mixed models with animal as a random effect, evidence for robust separation among objective FAMACHA© classes was limited. Among the individual BIA-related variables examined, only n_scans_180khz showed a statistically significant overall class effect after multiple-testing correction (chi-square = 23.92, *p* = 0.000026, false-discovery-rate-adjusted *p* = 0.000546). Estimated marginal means for this variable increased from 11.18 in class 1 and 11.41 in class 2 to 14.70 in class 3 and 17.67 in class 4, indicating a clear upward shift in higher-risk groups.

Post hoc comparisons confirmed that the main separation in n_scans_180khz occurred between the lower-risk and higher-risk classes. Significant Tukey-adjusted contrasts were observed for class 1 versus class 3, class 1 versus class 4, class 2 versus class 3, and class 2 versus class 4, whereas classes 1 and 2 did not differ from one another and classes 3 and 4 were also not significantly different. Thus, the strongest repeated-measures signal in the updated inferential analysis was concentrated in scan-count behavior at 180 kHz rather than in the core impedance means themselves.

Other BIA variables showed weaker or only borderline evidence of class separation. rs_sd_100khz approached significance in the overall mixed model, suggesting a possible but unstable class effect in resistance variability. phase_angle_180khz_deg and rs_mean_100khz showed smaller nonsignificant trends. After false-discovery-rate correction across variables, none of these effects remained significant. Overall, the repeated-measures mixed-model results indicate that once within-animal dependence is modeled directly, most BIA variables do not show strong or consistent class-level separation across the four objective FAMACHA© categories. Detailed assumption testing, global group-comparison test selection, and variable-level inferential results are provided in Supplementary Table S1.

### 3.4. Frequency-Wise Mixed-Model Analysis

To determine whether frequency-dependent behavior differed systematically across objective FAMACHA© classes, repeated-measures mixed models were also fit for grouped BIA families spanning 50, 100, and 180 kHz. Across these models, no frequency main effect, FAMACHA© main effect, or FAMACHA© × frequency interaction remained significant after false-discovery-rate correction. The strongest unadjusted evidence was observed for resistance standard deviation, where both the frequency effect and the FAMACHA© effect approached significance. Phase angle showed weaker, nonsignificant trends, and no family demonstrated a compelling interaction between FAMACHA© and frequency.

Pairwise frequency contrasts were also nonsignificant after multiple-testing correction. Some nominal differences were observed in specific within-class contrasts, but these did not survive adjustment. Taken together, the frequency-wise analysis suggests that frequency-related differences in the present dataset were modest and did not provide strong independent discrimination across objective FAMACHA© classes.

### 3.5. Correlation of BIA-Derived Variables with Packed Cell Volume

Exploratory Spearman correlation analysis identified weak negative associations between selected BIA-derived variables and PCV. Phase angle at 180 kHz showed a correlation of ρ = −0.171 (*n* = 177; unadjusted *p* = 0.023; animal-clustered bootstrap 95% CI: −0.292 to −0.049), while mean reactance at 180 kHz showed ρ = −0.153 (*n* = 177; unadjusted *p* = 0.042; 95% CI: −0.285 to −0.013). Although these associations reached nominal significance, neither remained statistically significant after Benjamini–Hochberg correction across 57 correlations (adjusted *p* = 0.358 for both). The reported confidence intervals account for animal-level clustering but are not adjusted for multiple comparisons. Overall, these findings indicate that the observed associations were weak and exploratory and do not establish a robust independent relationship between individual BIA variables and anemia severity. The repeated-measures mixed-effects analyses therefore provide the primary inferential basis for interpreting class-related differences. Complete correlation coefficients and unadjusted *p*-values are presented in Supplementary Table S2.

### 3.6. Binary Classification Performance

Binary classification of low-risk versus high-risk objective FAMACHA© was the strongest predictive task in the machine-learning analysis. Among all model and feature-block combinations, the best-performing model was logistic regression using the Full_without_PCV feature block. This model achieved a balanced accuracy of 0.707, macro F1 of 0.677, weighted F1 of 0.840, receiver operating characteristic area under the curve of 0.841, average precision of 0.529, and Cohen’s kappa of 0.357. Bootstrap confidence intervals for this best binary model were stable, with receiver operating characteristic area under the curve ranging from 0.760 to 0.910, balanced accuracy from 0.598 to 0.812, macro F1 from 0.580 to 0.768, and average precision from 0.338 to 0.711.

Feature-block comparison showed that integrated predictor sets outperformed BIA-only models. The best BIA-only binary model, also logistic regression, achieved a balanced accuracy of 0.648, macro F1 of 0.602, and receiver operating characteristic area under the curve of 0.643. Addition of site, weather, and fecal variables improved discrimination, with the strongest overall performance observed in the Full_without_PCV block. Thus, while BIA alone carried useful signal, predictive performance improved when impedance-derived variables were interpreted alongside contextual and biological covariates.

Other binary models generally performed below the best logistic model. The strongest non-logistic integrated classifier was the multilayer perceptron under the BIA_plus_site_plus_other block, with a macro F1 of 0.612, although its balanced accuracy remained below that of the best logistic framework. Random forest achieved relatively high probability discrimination in the Full_without_PCV block, with a receiver operating characteristic area under the curve of 0.838, but this did not translate into superior threshold-based classification performance. Overall, the binary analyses indicate that discrimination of low risk versus high-risk animals was feasible, but strongest when BIA was combined with environmental and parasitological information.

### 3.7. Three-Class Classification Performance

Predictive performance declined when the outcome was expanded from binary to three classes. The best three-class model was random forest using the BIA_raw_only block, with balanced accuracy of 0.429, macro F1 of 0.435, weighted F1 of 0.494, quadratic weighted kappa of 0.272, and macro one-versus-rest area under the curve of 0.653. Although this result suggests that raw BIA variables retained some class structure, the overall level of performance remained modest. Other leading three-class models yielded similarly limited performance, indicating that once the prediction task moved beyond low-risk versus high-risk discrimination, overlap among adjacent anemia-related states became substantial.

### 3.8. Four-Class Classification Performance

The full four-class objective FAMACHA© classification task was the weakest predictive setting. The best-performing four-class model was random forest under the BIA_plus_site_plus_other block, with balanced accuracy of 0.316, macro F1 of 0.321, weighted F1 of 0.495, quadratic weighted kappa of 0.225, and macro one-versus-rest area under the curve of 0.682. Several other four-class models performed similarly, but none approached the discrimination observed in the binary setting. The relatively poor performance of the four-class classification models should be interpreted in the context of the substantial class imbalance, particularly the limited representation of the most severe objective FAMACHA category (class 4; *n* = 3). This restricted sample size limits the ability of machine-learning models to learn reliable patterns associated with severe anemia and introduces uncertainty into class-specific performance estimates. Therefore, the observed reduction in multiclass performance may reflect both overlapping physiological characteristics among adjacent FAMACHA categories and insufficient representation of the severe-anemia category. These findings do not support reliable discrimination across all four severity classes under the present sampling conditions. Complete classification performance across binary, three-class, and four-class FAMACHA© prediction tasks, including all model families and feature blocks, is provided in Supplementary Table S3.

### 3.9. Regression Performance for Packed Cell Volume Prediction

Regression models showed that BIA and contextual features captured some continuous signal related to PCV, but overall predictive strength remained modest. The best regression model was RandomForest_regression under the Full_without_PCV block, with root mean squared error of 4.025, mean absolute error of 3.325, and *R*^2^ = 0.174. This was the strongest overall configuration, indicating that the integrated feature space explained a limited but non-negligible proportion of PCV variance.

Among non-integrated configurations, random forest using BIA_raw_only achieved root mean squared error of 4.311 and *R*^2^ = 0.053 whereas random forest using BIA_raw_plus_derived achieved root mean squared error of 4.360 and *R*^2^ = 0.031. These comparisons show that raw and derived BIA measurements alone captured only weak PCV-related structure, whereas the inclusion of contextual variables provided measurable improvement. Other regression approaches generally performed worse than random forest, and several weaker configurations yielded negative *R*^2^ values, indicating poor fit to the observed hematologic variation.

### 3.10. Variable Importance and Incremental Value of Integrated Feature Blocks

Permutation-importance analysis clarified the relative contribution of BIA and non-BIA variables in the best-performing predictive models. For the best binary classifier, the highest-ranking variables included fecal_count_numeric, avg_rainfall_in, avg_wind_speed_mph, xc_sd_100khz, and avg_relative_humidity_pct. Additional important contributors included phase_angle_50khz_deg, phase_angle_50_to_100_ratio, and rs_sd_180khz. The complete permutation-based variable importance ranking for the best-performing binary FAMACHA© classification model is provided in Supplementary Table S4. For the best regression model predicting PCV, the highest-ranking variables were avg_rainfall_in, fecal_count_numeric, rs_sd_mean_across_freq, phase_angle_180khz_deg, and n_scans_180khz, followed by other higher-frequency and variability-based impedance variables.

These rankings indicated that both BIA-derived and non-BIA variables contributed to model behavior. However, permutation importance alone does not establish whether the integrated framework provides measurable improvement over reduced predictor sets. Therefore, a formal ablation analysis was conducted to quantify the incremental value of BIA, fecal, weather/THI, raster-derived environmental, and full multimodal feature integration for binary objective FAMACHA© risk classification.

### 3.11. Raster-Derived Environmental Context of the Sampling Site

**Figure 3:**
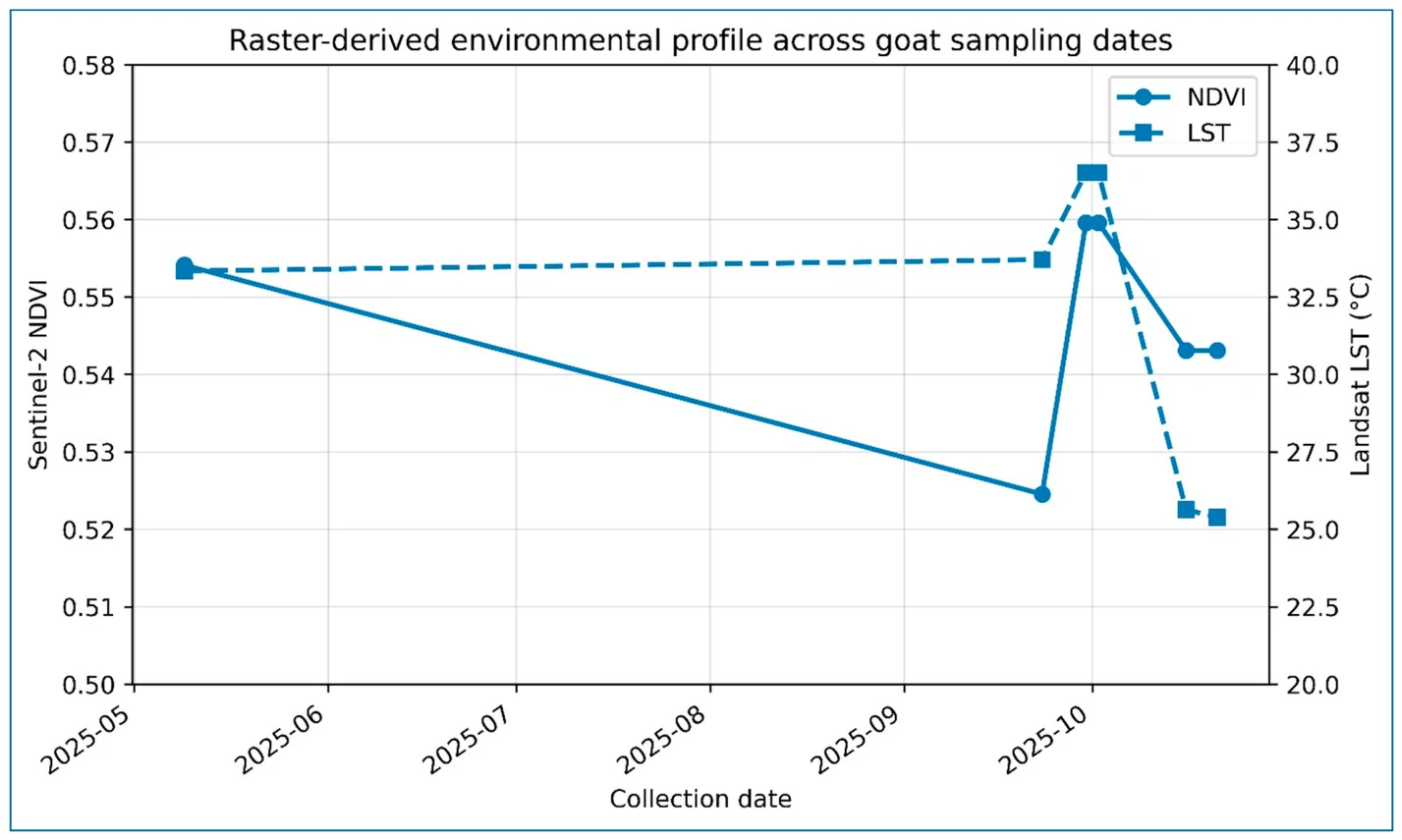
Raster-derived NDVI and land surface temperature across goat sampling dates. NDVI was extracted from Sentinel-2 surface reflectance using a ±7-day window around each sampling date, and land surface temperature was extracted from Landsat 8/9 surface temperature products using a ±16-day window. Values were extracted using a 50 m buffer around the final sampling coordinate.

Raster-derived environmental covariates provided additional site- and date-specific context for the multimodal anemia-monitoring framework. Across the six sampling dates, Sentinel-2-derived NDVI ranged from 0.525 to 0.560, indicating moderate and relatively stable vegetation activity within the 50 m extraction buffer. Landsat-derived land surface temperature showed greater temporal variation, ranging from 25.39 °C to 36.52 °C. The highest LST values occurred around 30 September and 2 October, whereas lower values were observed during the 16 October and 21 October sampling periods. Static site-level descriptors showed that the sampling coordinate was located at approximately 141.13 m elevation with a mean slope of 2.74°, and the dominant NLCD land-cover class was cultivated crops. Because the study was conducted at a single geographical location, elevation, slope, and land-cover classification were reported exclusively as descriptive site characteristics and were not included in any machine-learning models. Accordingly, no independent predictive contribution was attributed to these static geographical variables. Temporal variation in NDVI and land surface temperature across sampling dates is shown in Figure 3.

### 3.12. Incremental Value of Multimodal Feature Integration

**Figure 4:**
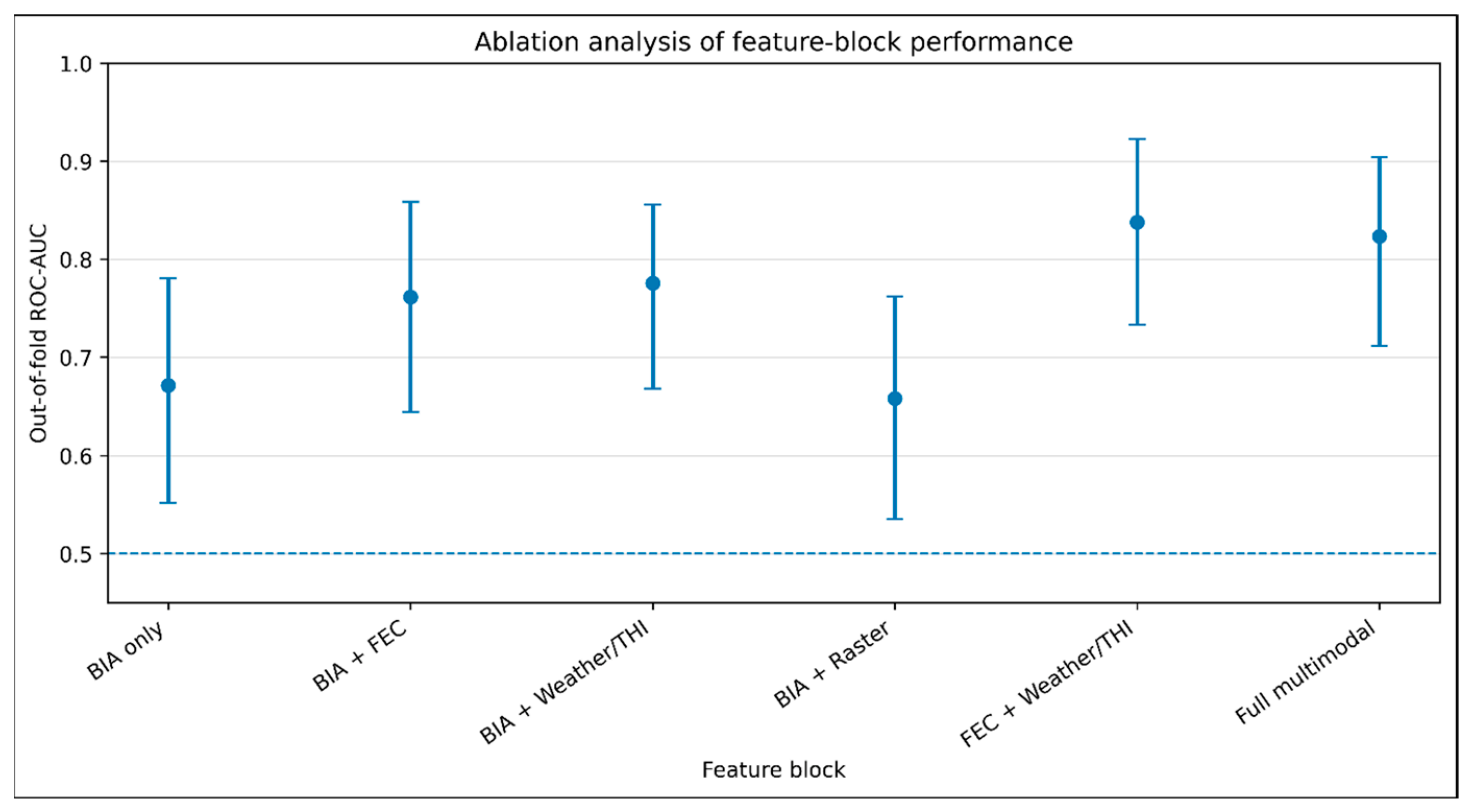
Ablation analysis of feature-block performance for binary objective FAMACHA© risk classification. Points show animal-grouped cross-validated ROC-AUC values from balanced logistic regression models trained on reduced and integrated feature blocks. Error bars indicate bootstrap 95% confidence intervals. The analysis shows limited discrimination from BIA-only features, improved performance after addition of fecal and weather/THI variables, and strong performance from the full multimodal feature block. The fecal count plus weather/THI block achieved the highest ROC-AUC, indicating that parasitological and environmental variables were dominant contributors in the present dataset. The classification analysis included 181 labeled animal-by-date-by-site observations from 79 goats. Abbreviations: BIA, bioelectrical impedance analysis; FEC, fecal egg count; THI, temperature-humidity index; ROC-AUC, area under the receiver operating characteristic curve; CI, confidence interval.

A standardized ablation analysis was conducted to evaluate whether multimodal feature integration improved binary objective FAMACHA© risk classification relative to reduced predictor sets. Balanced logistic regression was consistently applied across six feature blocks using animal-grouped five-fold cross-validation to minimize information leakage between training and testing observations. The BIA-only model achieved a ROC-AUC of 0.672, balanced accuracy of 0.578, F1-score of 0.267, and Brier score of 0.212. Incorporating fecal egg count (FEC) into the BIA feature block increased ROC-AUC to 0.761, whereas combining BIA with weather and temperature-humidity index (THI) variables achieved a ROC-AUC of 0.775. The BIA plus raster-derived environmental feature block achieved a ROC-AUC of 0.658, indicating that the inclusion of raster-derived covariates did not improve discrimination relative to BIA-only prediction in the present single-site dataset. Notably, the FEC plus weather/THI feature block achieved the highest ROC-AUC of 0.838, whereas the complete multimodal model achieved a ROC-AUC of 0.824, balanced accuracy of 0.691, F1-score of 0.419, and the lowest Brier score of 0.135. These findings demonstrate that parasitological and weather/THI variables provided the strongest discriminatory performance, whereas BIA-only prediction showed comparatively limited effectiveness. Importantly, the higher performance of the complete multimodal model relative to BIA alone cannot be interpreted as evidence of an independent incremental benefit attributable to BIA. The performance of the original six feature blocks is presented in Figure 4.

To further investigate the incremental predictive contribution of BIA, a supplementary matched feature-block sensitivity analysis was performed. The analysis included 181 observations with available objective FAMACHA classifications from 79 goats. Two balanced logistic regression models were compared using identical animal-grouped five-fold cross-validation splits: a conventional baseline model containing FEC and weather/THI predictors and an augmented model incorporating the same conventional predictors together with BIA-derived features. The baseline model achieved an out-of-fold ROC-AUC of 0.853, compared with 0.870 for the BIA-augmented model (ΔAUC = 0.016; animal-clustered bootstrap 95% CI: −0.052 to 0.082). Although the addition of BIA produced a small numerical improvement, the confidence interval included zero, indicating that a statistically reliable incremental benefit was not demonstrated. When BIA scan-count features were excluded, the estimated improvement was further reduced (ΔAUC = 0.001; 95% CI: −0.073 to 0.069). A complete-case sensitivity analysis restricted to 76 observations with measured FEC yielded ROC-AUC values of 0.723 and 0.770 for the conventional and BIA-augmented models, respectively (ΔAUC = 0.048; 95% CI: −0.107 to 0.213). Furthermore, evaluation across 20 alternative animal-grouped cross-validation partitions demonstrated variability in the estimated incremental contribution of BIA, with ΔAUC ranging from −0.140 to 0.035. These results indicate that the observed numerical improvement was sensitive to feature selection, missing FEC measurements, and data partitioning. Overall, the supplementary findings do not establish a reproducible incremental improvement in anemia-risk discrimination from adding BIA beyond conventional parasitological and environmental predictors. Detailed matched-model comparisons and cross-validation sensitivity results are presented in Supplementary Tables S7 and S8, respectively.

Paired bootstrap comparisons further quantified the incremental improvement of the full multimodal model relative to reduced feature blocks. The full multimodal model significantly improved ROC-AUC compared with BIA-only features, with a mean ΔAUC of 0.150 and 95% confidence interval of 0.072 to 0.232 (*p* < 0.001). The full model also improved over BIA plus fecal count, with ΔAUC of 0.061 and 95% confidence interval of 0.020 to 0.110 (*p* = 0.008), and over BIA plus raster-derived variables, with ΔAUC of 0.164 and 95% confidence interval of 0.065 to 0.260 (*p* = 0.004). Improvement over BIA plus weather/THI was borderline and not statistically significant at the 0.05 level (ΔAUC = 0.047; 95% CI, −0.003 to 0.096; *p* = 0.056). The full model did not improve over the fecal count plus weather/THI block (ΔAUC = −0.013; 95% CI, −0.124 to 0.093; *p* = 0.844). These findings indicate that parasitological and weather/THI predictors accounted for the strongest discriminatory performance in the original ablation analysis. However, because the complete multimodal feature block included BIA and additional environmental predictors, this comparison alone could not isolate the independent incremental contribution of BIA.

In the supplementary matched feature-block analysis, the conventional fecal egg count plus weather/THI model achieved an out-of-fold ROC-AUC of 0.853, compared with 0.870 after adding BIA-derived features (ΔAUC = 0.016; animal-clustered bootstrap 95% CI: −0.052 to 0.082). Although the BIA-augmented model showed a small numerical improvement, the confidence interval included zero. Excluding BIA scan-count features reduced the difference to ΔAUC = 0.001 (95% CI: −0.073 to 0.069). In a complete-case sensitivity analysis restricted to 76 observations with measured fecal egg counts, the baseline and BIA-augmented models achieved ROC-AUC values of 0.723 and 0.770, respectively (ΔAUC = 0.048; 95% CI: −0.107 to 0.213). Across 20 alternative animal-grouped cross-validation splits, ΔAUC ranged from −0.140 to 0.035, indicating sensitivity to data partitioning. These supplementary findings did not demonstrate a statistically reliable or reproducible incremental improvement from adding BIA.

Threshold analysis was used to evaluate the practical decision-support behavior of the full multimodal model. A screening-oriented threshold of 0.13 identified 20 of 24 high-risk observations, corresponding to sensitivity of 83.3%, specificity of 71.3%, balanced accuracy of 77.3%, precision of 30.8%, and F1-score of 0.449. Descriptive 95% Wilson confidence intervals were 64.1–93.3% for sensitivity, 63.8–77.8% for specificity, and 20.9–42.8% for positive predictive value. These approximate observation-level intervals do not account for within-animal clustering and should therefore be interpreted cautiously rather than as independently validated screening-performance estimates. This threshold produced four false negatives and 45 false positives. From a clinical perspective, the positive predictive value of 30.8% indicates that only 20 of the 65 observations flagged as high risk corresponded to the higher-risk reference category, while 45 represented false-positive classifications. These false positives could increase animal handling, follow-up examinations, and confirmatory testing requirements if the model were implemented in practice. Conversely, the four false-negative observations indicate a potential risk of missed anemia-related events and delayed clinical assessment or intervention. Importantly, these classification counts represent animal-by-date-by-site analytical observations rather than necessarily distinct animals. Therefore, the present threshold should be considered exploratory and should not be used independently to guide treatment decisions without clinical confirmation and prospective validation. However, because the threshold-selection procedure was not independently validated, the sensitivity and specificity estimates obtained at the 0.13 threshold may be optimistic and require confirmation through nested cross-validation or an independent validation dataset.

## 4. Discussion

The analysis provides a more constrained and realistic view of the role of bioelectrical impedance analysis in anemia-related monitoring in goats. Across the dataset, objective and field FAMACHA© classifications showed strong agreement, binary low-risk versus high-risk prediction was clearly more successful than three-class or four-class prediction, and multimodal models outperformed BIA-only configurations for both classification and regression. At the same time, repeated-measures mixed-model analysis showed limited direct class separation for most individual BIA variables once within-animal dependence was modeled explicitly. This distinction is important. It suggests that BIA contributes useful signals in predictive frameworks, but that the evidence for strong standalone variable-level separation is limited in the present dataset.

One of the clearest results was the strong agreement between field and objective FAMACHA©. This supports the continued practical value of field scoring and is consistent with earlier validation studies showing that FAMACHA© can function effectively as an on-farm tool for identifying anemia-related parasite burden in sheep and goats [5,6,16,18]. At the same time, the high but imperfect agreement justifies the use of PCV-derived objective FAMACHA© as the reference endpoint for model development [5,6,17]. Thus, the present findings do not undermine field FAMACHA©; rather, they indicate that field scoring remains useful for management, whereas objective classification is more appropriate for data-driven modeling and quantitative evaluation.

The descriptive patterns in PCV and fecal egg count were biologically coherent and consistent with the known pathophysiology of *Haemonchus contortus*, a blood-feeding nematode capable of producing clinically important anemia, production loss, and mortality in small ruminants [8,9,10]. However, once BIA variables were analyzed using repeated-measures mixed models, most individual impedance measures did not show strong class-level separation. Only scan count at 180 kHz remained significant after multiple-testing correction, whereas phase angle, resistance variability, and related higher-frequency features showed only modest or borderline effects. This is a more conservative result than the earlier nonparametric screening approach and indicates that much of the apparent separation seen at the descriptive level is weakened when within-animal correlation is handled directly.

This finding has important implications for interpretation. It suggests that in the present dataset, BIA does not behave as a strong standalone marker that cleanly separates all objective FAMACHA© classes at a variable level. Rather, its value is more evident when used as part of an integrated predictive framework. That conclusion is reinforced by the machine-learning results. Binary classification performed substantially better than three-class or four-class prediction, which is biologically plausible because the contrast between lower-risk and higher-risk animals are easier to capture than finer distinctions among adjacent intermediate states. This pattern is also consistent with the practical logic of FAMACHA© itself, which is most often used to identify animals requiring management attention rather than to resolve highly granular severity categories [16,18,19].

The ablation analysis provides an important distinction between the predictive performance of the complete multimodal framework and the independent contribution of BIA-derived measurements. Although the complete multimodal model significantly outperformed BIA-only prediction, it did not significantly improve discrimination compared with the fecal egg count plus weather/THI feature block (ΔAUC = −0.013; 95% CI: −0.124 to 0.093; *p* = 0.844). This finding indicates that parasitological and environmental predictors accounted for the strongest discriminatory performance in the present dataset. Furthermore, because the complete model included both BIA and raster-derived environmental variables, the original comparison could not isolate the incremental contribution attributable specifically to BIA. To address this limitation, a supplementary matched feature-block analysis compared conventional predictors with the identical predictor set augmented exclusively with BIA-derived measurements. Although the augmented model showed a small numerical increase in ROC-AUC, the estimated improvement was imprecise, and its confidence interval included zero. Excluding scan-count variables almost completely eliminated the observed difference. These findings suggest that the independent predictive value of BIA remains unestablished rather than demonstrating a reproducible additional benefit. Interpretation of the supplementary analysis also requires caution because fecal egg counts were missing for 105 of the 181 labeled observations, and the analysis was reconstructed from an archived dataset that did not reproduce the exact original modeling pipeline. Consequently, the predictive performance of the multimodal framework should not be interpreted as evidence that BIA independently improves anemia-risk classification beyond conventional parasitological and environmental predictors. The principal contribution of the present study is therefore the systematic evaluation of BIA within an environment-aware multimodal monitoring framework, with its potential additional predictive value remaining an important subject for further investigation.

The stronger performance of the FEC and weather/THI feature block is biologically plausible. Fecal egg count provides an indirect indicator of gastrointestinal nematode burden, while environmental conditions influence parasite development, survival, and transmission [2,8] and may also affect host physiological responses [8,9,20,22]. In contrast, BIA measures the electrical characteristics of biological tissues [25,26,27], which are influenced by several interacting physiological processes rather than anemia alone. Consequently, impedance-derived measurements may capture physiological variation associated with anemia risk without providing sufficient disease-specific information to improve discrimination beyond established parasitological and environmental predictors. These findings suggest that FEC and weather/THI variables were the dominant predictive components in the present dataset, whereas the additional contribution of BIA remains unconfirmed. The practical significance of BIA may therefore lie in its potential role as a complementary physiological sensing modality for longitudinal monitoring rather than as an independent diagnostic tool. Establishing whether BIA provides additional predictive or operational benefits will require further evaluation under independent field conditions.

The permutation–importance analysis provides further context regarding the relative influence of BIA-derived and non-BIA predictors. Fecal egg count and weather-related variables, including rainfall, wind speed, and relative humidity, were among the highest-ranking predictors in the binary classification model, alongside selected impedance-derived features. A similar pattern was observed in PCV regression, where parasitological and environmental variables remained prominent. However, the importance of individual impedance features within a fitted model should not be interpreted as evidence of their independent incremental predictive value. When considered alongside the ablation analysis, these findings indicate that parasitological and weather/THI variables provided the principal discriminatory information in the present dataset. The complete multimodal feature set, which included BIA measurements, did not significantly improve discrimination beyond the fecal egg count and weather/THI model. Consequently, BIA cannot presently be justified as the primary predictive component of the proposed monitoring framework. Instead, impedance-derived measurements should be regarded as potentially supplementary physiological indicators whose independent predictive contribution requires further validation.

The raster-derived environmental covariates further support the interpretation that anemia-related monitoring in goats should be viewed within an environmental and management context rather than through BIA alone. NDVI remained moderately stable across sampling dates, suggesting relatively consistent vegetation activity at the site, whereas land surface temperature varied substantially across the study period. This variation is biologically relevant because thermal conditions, vegetation status, pasture environment, and moisture-related factors can influence parasite exposure, animal hydration, tissue conductivity, and physiological stress [2,8,25,26]. The interpretation of raster-derived environmental measurements is constrained by the single-site study design. Static geographical variables, including elevation, slope, and land-cover classification, were used exclusively for environmental characterization and were not incorporated into the predictive models. Although NDVI and LST exhibited variation across sampling dates, these measurements represent temporal environmental changes within a single geographical location rather than independent spatial differences. Consequently, the present findings do not establish the spatial generalizability of these environmental predictors. Future investigations involving multiple farms and geographically distinct production environments will be necessary to evaluate their broader predictive relevance.

The correlation analysis further highlights the limited physiological specificity of BIA-derived measurements for anemia assessment. Although selected higher-frequency impedance features showed weak nominal associations with packed cell volume (PCV), none remained statistically significant after correction for multiple comparisons, and the observed electrical changes cannot be attributed specifically to anemia. The correlations were weak and do not establish that the observed electrical changes were directly attributable to anemia. Resistance, reactance, and phase angle are influenced by tissue hydration, electrolyte distribution, cellular composition, and membrane-related electrical properties [25,27]. Consequently, changes in these measurements may arise from several physiological conditions independently of anemia severity. For example, dehydration can alter tissue conductivity through changes in fluid distribution, while differences in body composition and physiological stress may influence impedance responses. These overlapping effects complicate the interpretation of BIA-derived variables as anemia-specific indicators. Therefore, the observed relationships should be considered associations between tissue electrical properties and anemia-related physiological status rather than evidence of a direct or disease-specific electrical signature of anemia. The regression findings further emphasize this point. PCV prediction was feasible but explained variance remained modest. An *R*^2^ of 0.174 indicates that the integrated predictors captured some meaningful hematologic structure but did not provide high-fidelity reconstruction of PCV. From a practical perspective, these findings suggest that the framework may warrant further investigation as a supplementary screening or triage approach rather than a method for direct hematological estimation or independent clinical decision-making; however, its limited positive predictive value, potential for missed higher-risk observations, and lack of prospective external validation currently preclude recommendations for routine clinical implementation.

That distinction matters for technology translation. In smart livestock systems, practical monitoring value often lies in improving treatment prioritization or repeated surveillance under field conditions rather than in perfectly reproducing laboratory measurements. The findings also highlight the distinct but complementary roles of conventional clinical assessments, parasitological indicators, and bioelectrical impedance analysis (BIA) in anemia-risk monitoring. Packed cell volume (PCV) provides a direct hematological measure of anemia severity and served as the physiological reference for deriving objective FAMACHA classifications in the present study [5,6,39]. Field-based FAMACHA scoring offers a practical, low-cost approach for evaluating anemia-related risk through ocular mucous membrane color, although its interpretation may be influenced by observer experience and biological variability [5,6,16,17,18]. Fecal egg count provides an indirect indicator of gastrointestinal nematode burden through parasite egg shedding and contributes important parasitological information, although it does not directly quantify anemia severity [8,9,20,22]. In contrast, BIA measures the electrical properties of biological tissues, reflecting variations in fluid distribution, tissue composition, and membrane-related characteristics rather than anemia-specific hematological changes [25,26,27,28,29]. Its potential value lies in providing additional physiological measurements that could complement established clinical and parasitological assessments during repeated monitoring [27,35,38,40]. However, BIA-derived signals are nonspecific and may be influenced by hydration status, body condition, and physiological stress [25,27]. Furthermore, the present study demonstrated limited discrimination using BIA alone, while the complete multimodal model did not significantly outperform the fecal egg count and weather/THI predictor set. Consequently, the independent incremental value of BIA remains unestablished, and its potential application should be restricted to a supplementary sensing role until further validation is available. BIA therefore cannot replace PCV for hematological assessment, FAMACHA for practical field-based anemia evaluation, or fecal egg counting for parasitological assessment. Instead, its future utility may lie in contributing complementary physiological information to multimodal decision-support systems [35,38,40], with clinical interpretation and management decisions remaining supported by established diagnostic and monitoring methods [5,9,16,18,20,39].

**Figure 5:**
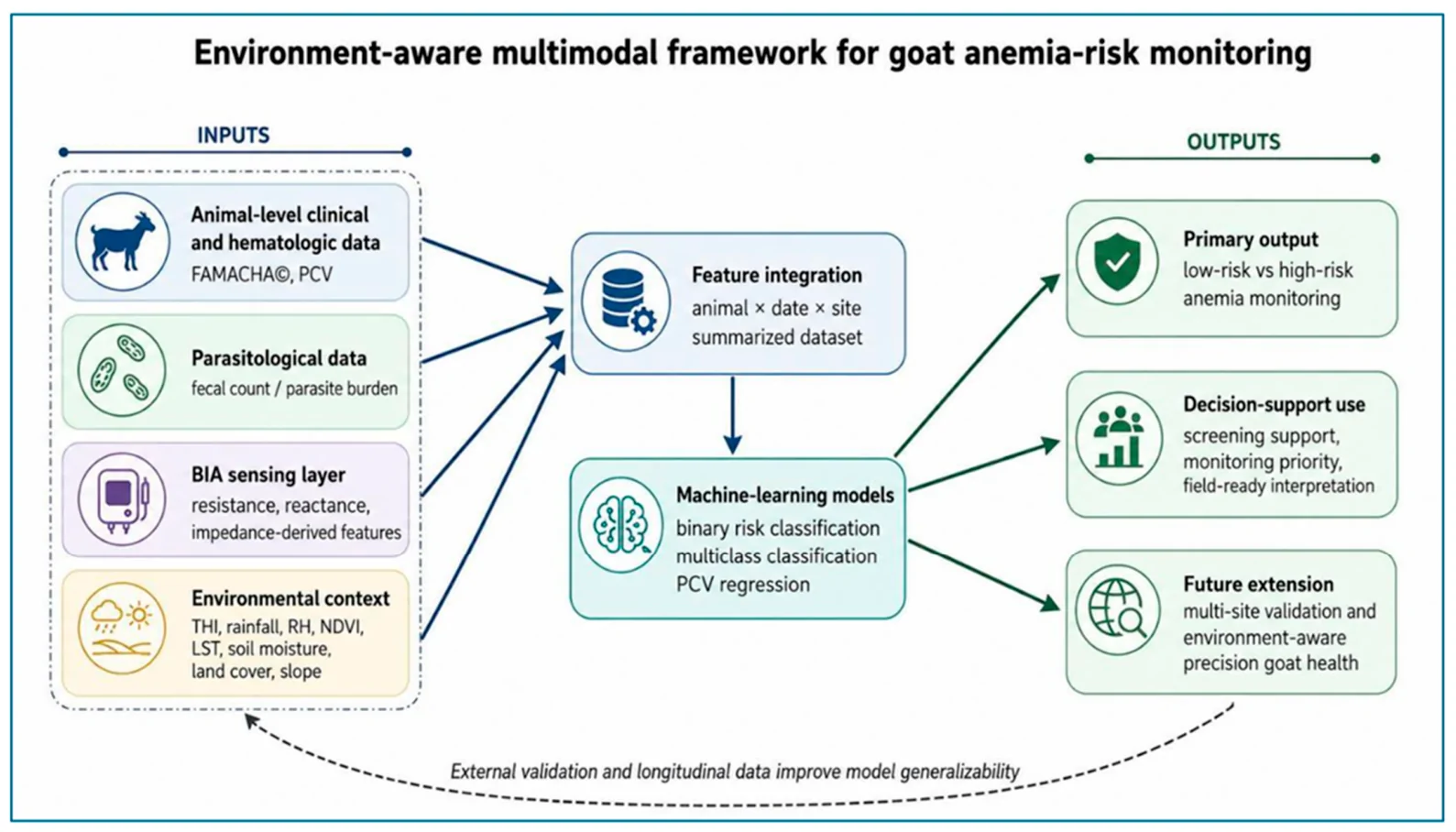
Conceptual framework for environment-aware multimodal goat anemia-risk monitoring. The framework illustrates how animal-level clinical and hematological reference data, parasitological indicators, BIA-derived sensing features, and environmental covariates can be integrated into machine-learning models for anemia-risk classification, PCV regression, and field-oriented decision-support development. Although the proposed framework was evaluated using internal animal-grouped cross-validation, its generalizability remains unestablished. Therefore, independent longitudinal datasets collected from different goat breeds, farms, and management conditions are required to evaluate model robustness, generalizability, and practical applicability before broader field implementation.

To synthesize the analytical structure and applied interpretation of the present study, a conceptual framework was developed to illustrate how animal-level clinical data, parasitological indicators, BIA-derived sensing features, and environmental covariates can be integrated into machine-learning models for goat anemia-risk monitoring (Figure 5). This framework emphasizes that BIA should be interpreted as a complementary sensing layer within a broader multimodal decision-support system rather than as a standalone diagnostic replacement for FAMACHA© scoring, PCV, or fecal-based parasite assessment.

The present study also has important limitations. First, the distribution of objective FAMACHA categories was markedly imbalanced, with the most severe category (class 4) represented by only three observations. This imbalance limits the statistical precision of comparisons involving severe anemia and reduces the number of examples available for learning and evaluating class-specific patterns. Although animal-grouped cross-validation was implemented to prevent information leakage from repeated measurements, this approach cannot overcome the limited representation of the severe category. Furthermore, the stronger performance of binary classification should not be interpreted as evidence of reliable identification of the most severe anemia cases independently, because classes 3 and 4 were combined into a single high-risk category. Consequently, the present findings provide stronger support for distinguishing lower risk from higher-risk observations than for accurately differentiating individual anemia severity categories. Future studies should incorporate larger and more balanced datasets with adequate representation of severe-anemia observations to improve the reliability of multiclass prediction and assessment of severe-anemia detection.

The limited effective sample size and substantial class imbalance also warrant careful consideration when interpreting model performance. Although the analysis-ready dataset contained 181 labeled observations, these originated from only 79 goats, and the higher-risk categories were represented by 24 observations from 21 unique animals. Importantly, only three goats contributed class 4 observations, limiting the reliability of severe-anemia discrimination and multiclass performance estimates. The reconstructed animal-grouped cross-validation partitions contained only three to six high-risk observations per held-out fold, indicating that classification sensitivity could be substantially influenced by the correct or incorrect classification of a small number of animals. Furthermore, repeated observations obtained from different anatomical sites on the same date increased the number of analytical records without providing additional independent animals. Only 31 goats were observed on multiple collection dates, of which 11 exhibited changes in anemia-risk classification over time. The uneven temporal distribution of risk categories further complicates interpretation, as the initial collection date contributed a substantial number of low-risk observations but no high-risk observations. Although animal-grouped cross-validation reduced the risk of information leakage, it could not compensate for the limited number of independent high-risk animals or the restricted representation of severe anemia. Consequently, the reported sensitivity, multiclass discrimination, and overall predictive performance should be regarded as preliminary estimates requiring confirmation in larger longitudinal datasets with more balanced representation of anemia severity, additional independent animals, and external validation across different farms and management environments.

Second, the distinction between raw scan-level measurements and analysis-ready summarized observations must be recognized clearly. The raw dataset contained tens of thousands of scan rows, but inferential and predictive analyses were necessarily performed at the summarized animal-by-sampling-event level to avoid pseudo replication. Third, the present study was conducted using 81 Spanish intact male goats from a single study location, which limits the generalizability of the predictive models. Although animal-grouped cross-validation was implemented to prevent information leakage between training and testing observations from the same animals, all observations originated from the same breed and production environment. Therefore, the reported model performance represents internal validation and should not be interpreted as evidence of predictive reliability across different goat breeds, farms, or management conditions. Differences in animal genetics, age, sex, nutritional status, parasite exposure, climatic conditions, and husbandry practices may influence both physiological impedance measurements and relationships between parasitological indicators and anemia severity. These differences could affect model performance when applied to independent populations. In particular, sex- and age-related differences in body composition, hydration status, nutritional condition, and reproductive or other physiological states may influence tissue electrical properties and BIA responses [25,26,27,28,29]. Because the present study included only intact male goats of similar age, the effects of these factors on impedance measurements and anemia-risk prediction could not be independently evaluated.

Furthermore, the relative predictive contributions of BIA, fecal egg count, and environmental variables may differ across production systems. Future investigations should therefore prioritize independent external validation using longitudinal datasets collected from multiple herds, including male and female goats, different age groups and breeds, and diverse seasons, environmental conditions, and management systems. Such studies should evaluate discrimination, calibration, and classification performance before the proposed framework can be considered suitable for broader field application. In addition, raster-derived variables were extracted for a single final sampling coordinate; therefore, elevation, slope, and land-cover class were used descriptively rather than as independent spatial predictors.

Future studies involving geographically distinct farms should investigate whether temporally varying environmental indicators, such as NDVI, land surface temperature (LST), and soil moisture, together with site-specific characteristics, including land cover and topography, improve anemia-risk prediction beyond established parasitological and weather/THI predictors. In the present single-site study, static geographical variables were used exclusively for environmental characterization and were not included in the predictive models. Furthermore, because BIA measurements are influenced by multiple physiological processes, including hydration status, body composition, tissue conductivity, and environmental stress, the observed associations cannot be attributed specifically to anemia. An additional limitation concerns the reproducibility of BIA acquisition under field conditions. Variation in electrode placement, skin preparation, electrical contact, animal posture, hydration [25,26,27], and environmental conditions may influence impedance measurements, while formal within-session and between-session repeatability estimates were not available for the present analysis. Furthermore, the observed association involving scan count at 180 kHz may partly reflect measurement-acquisition variability rather than anemia-related physiological changes, emphasizing the need for standardized measurement protocols, documented quality-control criteria, instrument calibration, and prospective repeatability assessment in future studies.

Taken together, these findings support a cautious interpretation of BIA’s predictive value. Most impedance-derived variables did not demonstrate robust separation across objective FAMACHA categories under repeated-measures modeling, and the complete multimodal model did not significantly outperform the fecal egg count plus weather/THI model. Therefore, the independent incremental predictive benefit of BIA remains unestablished in the present dataset. Nevertheless, BIA may provide complementary physiological information that warrants further investigation within multimodal livestock health monitoring systems. Future research should prioritize larger and more balanced datasets, particularly with adequate representation of severe-anemia cases, longitudinal monitoring of repeated BIA measurements, and independent validation across different goat breeds, farms, and management conditions. Such investigations are necessary to determine whether BIA offers reproducible and clinically meaningful additional information before its integration into practical field-based decision-support systems.

## 5. Conclusions

This study evaluated bioelectrical impedance analysis within a multimodal framework for anemia-related monitoring in goats using objective FAMACHA© classification and packed cell volume as reference outcomes. Objective and field-based FAMACHA© classifications showed strong agreement, confirming that field scoring remains a useful practical tool while supporting the value of PCV-derived objective classification for analytical modeling. Among the predictive tasks examined, binary classification of low-risk versus high-risk animals was the most successful, outperforming both three-class and four-class models. However, the limited representation of the severe objective FAMACHA category (class 4; *n* = 3) restricts the reliability of multiclass classification and prevents firm conclusions regarding the model’s ability to distinguish severe anemia independently. Additional validation using larger and more balanced datasets is necessary before extending the framework to reliable classification across individual anemia severity categories.

Repeated-measures mixed-model analysis showed limited direct class separation for most individual BIA variables once within-animal dependence was accounted for, with the clearest signal observed for scan count at 180 kHz. The machine-learning results further demonstrated that parasitological and weather/THI variables provided the strongest discriminatory information, while the additional predictive benefit of BIA remained unconfirmed. These findings do not support positioning BIA as the primary predictor of anemia risk in the present dataset.

The addition of THI and raster-derived environmental covariates further supports the interpretation that BIA-based anemia monitoring is most appropriately developed as part of an environment-aware multimodal framework. Date-specific NDVI and LST provided temporal environmental context, while elevation, slope, and land cover described the local sampling environment. Regression models captured some continuous variation in packed cell volume, but predictive strength remained modest, indicating that the present framework is more suitable for risk stratification and monitoring support than for exact hematologic estimation. Taken together, these findings indicate that BIA is best interpreted not as a standalone replacement for conventional clinical or hematologic assessment, but as a complementary sensing component within multimodal smart-monitoring systems for goats. The ablation analysis strengthens the central conclusion of the study. BIA-only models showed limited discrimination, whereas feature blocks incorporating fecal and weather/THI variables produced substantially stronger predictions.

Although the complete multimodal model significantly outperformed BIA-only prediction, it did not demonstrate a significant improvement over the fecal egg count and weather/THI feature block. This finding indicates that parasitological and environmental variables were the principal contributors to anemia-risk discrimination in the present dataset, whereas the independent incremental predictive benefit of BIA was not established. This interpretation was further supported by a supplementary matched feature-block analysis, in which the addition of BIA to an otherwise identical conventional predictor set produced a small and statistically uncertain improvement in discrimination. Therefore, the present findings do not establish that BIA provides reproducible incremental predictive information beyond fecal egg count and weather/THI variables. Therefore, the primary contribution of this study is the systematic evaluation of BIA within an environment-aware multimodal monitoring framework rather than its validation as a standalone or primary predictor of anemia risk. Although BIA may provide complementary physiological information, its measurements are influenced by multiple factors, including hydration status, body composition, and physiological stress, limiting their specificity for anemia-related changes. Furthermore, the models were evaluated through internal animal-grouped cross-validation using a single goat breed at one study location, with limited representation of severe-anemia cases. Consequently, their generalizability across different breeds, farms, and management conditions remains uncertain. Future research should prioritize larger and more balanced longitudinal datasets, independent assessment of physiological confounding factors, and external validation across diverse production environments to determine whether BIA provides reproducible and clinically meaningful additional information beyond established clinical and parasitological assessment methods.

## Data Availability

As this is an ongoing study, the data are not publicly available at this time but may be made available by the corresponding author upon reasonable request.

## Supplementary Materials

The following supporting information can be downloaded at: https://www.mdpi.com/article/10.3390/ani16213441/s1. Table S1. Results of assumption testing and global group-comparison test selection for bioelectrical impedance-derived, scan-count, and biological variables across objective FAMACHA classes; Table S2. Spearman rank correlation coefficients (ρ) and associated *p*-values for the relationships between bioelectrical impedance analysis–derived variables and packed cell volume (PCV); Table S3. Classification performance across binary, three-class, and four-class FAMACHA© prediction tasks across model families and feature blocks; Table S4. Permutation-based variable importance rankings for the best-performing binary FAMACHA classification model. Importance values represent the mean decrease in model performance after random permutation of each feature, and the accompanying standard deviation reflects variability across repeated permutations; Table S5. Ablation performance of feature blocks for binary objective FAMACHA© risk classification; Table S6. Threshold-based decision-support performance of the full multimodal model for binary objective FAMACHA© risk classification; Table S7. Matched conventional versus BIA-augmented feature-block comparisons for binary objective FAMACHA anemia-risk classification; Table S8. Sensitivity of the matched incremental BIA effect to 20 alternative animal-grouped cross-validation partitions (full 181-observation cohort); Table S9. Animal-level representation, repeated-observation structure, and variation in objective FAMACHA anemia-risk categories; Table S10. Observed risk-class composition of the reconstructed five-fold animal-grouped cross-validation test partitions (*n* = 181 labeled observations from 79 goats); Figure S1. Bootstrap comparison of incremental ROC-AUC improvement for the full multimodal model relative to reduced feature blocks.
