# Supplementary material for: Assessing the Incremental Value of Environment-Aware Bioelectrical Impedance Sensing for Machine Learning-Based Anemia Risk Monitoring in Goats
Source: Animals (Basel). 2026 Oct 31;16(21):3441. doi: 10.3390/ani16213441 (PMC13762347; doi:10.3390/ani16213441)
Supplement: Supplementary file 1 [file animals-16-03441-s001.zip › animals-4642973-supplementary.pdf]

Article

Assessing the Incremental Value of Environment-Aware Bioelectrical Impedance Sensing for Machine Learning-Based Anemia Risk Monitoring in Goats

Ramya Kota [1], Shreya Neelagiri [1], Goutham Kumar Isai [1], Davia Brown [1], Jayanta Banik [1], Andrea Villa Rubio [1], Phaneendra Batchu [1], David Shapiro-Ilan [2], Cristina Pisani [2], Aftab Siddique [1,*], Ajit K. Mahapatra [1], Sudhanshu S. Panda [3] and Thomas H. Terrill [1]

[1] Department of Agricultural Sciences, Fort Valley State University, Fort Valley, 31030, GA, USA; rkota@wildcat.fvsu.edu (R.K.); sneelagi@wildcat.fvsu.edu (S.N.); gisai@wildcat.fvsu.edu (G.K.I.); dbrow212@wildcat.fvsu.edu (D.B.); jayanta.banik@fvsu.edu (J.B.); arubiovi@wildcat.fvsu.edu (A.V.R.); phaneendra.batchu@fvsu.edu (P.B.); mahapatraa@fvsu.edu (A.K.M.); terrillt@fvsu.edu (T.H.T.)

[2] USDA-ARS, Fruit and Tree Nut Research Station, Byron, GA, 31008, USA; david.shapiro@usda.gov (D.S.-I.); cristina.pisani@usda.gov (C.P.)

[3] Institute for Environmental Spatial Analysis, University of North Georgia, 3820 Mundy Mill Road, Oakwood, GA 30566, USA; sudhanshu.panda@ung.edu

* Correspondence: aftab.siddique@fvsu.edu

Table S1. *Results of assumption testing and global group-comparison test selection for bioelectrical impedance-derived, scan-count, and biological variables across objective FAMACHA classes.*

Variable	shapiro_p_by_class	levene_p	anova_p	kruskal_p	recommended_test
n_scans_100khz	1:0.0017; 2:0.0; 3:0.0064; 4:1.0	0.523663087	0.265826163	0.255766287	Kruskal-Wallis
n_scans_180khz	1:0.0; 2:0.0; 3:0.0012; 4:0.3914	0.066371492	6.17E-05	0.006190567	Kruskal-Wallis
n_scans_50khz	1:0.0021; 2:0.0; 3:0.0064; 4:NA	0.615004617	0.426570601	0.285244945	Kruskal-Wallis
phase_angle_100_minus_50	1:0.0179; 2:0.048; 3:0.0003; 4:NA	0.145344632	0.817974898	0.485287337	Kruskal-Wallis
phase_angle_100_to_180_ratio	1:0.0; 2:0.0; 3:0.0; 4:0.0049	0.725203782	0.765033302	0.774836222	Kruskal-Wallis
phase_angle_100khz_deg	1:0.3224; 2:0.2012; 3:0.147; 4:0.7274	0.694328978	0.347394119	0.360490417	ANOVA
phase_angle_180_minus_100	1:0.0598; 2:0.0002; 3:0.0033; 4:0.086	0.861114292	0.81690845	0.659853523	Kruskal-Wallis
phase_angle_180_minus_50	1:0.0405; 2:0.0321; 3:0.1174; 4:NA	0.806708686	0.716382681	0.806966357	Kruskal-Wallis
phase_angle_180khz_deg	1:0.0002; 2:0.0175; 3:0.7238; 4:0.76	0.939645227	0.226075052	0.195950872	Kruskal-Wallis
phase_angle_50_to_100_ratio	1:0.0; 2:0.0; 3:0.0; 4:NA	0.350097568	0.367980658	0.582544119	Kruskal-Wallis
phase_angle_50_to_180_ratio	1:0.0; 2:0.002; 3:0.0; 4:NA	0.612680308	0.457375037	0.716583204	Kruskal-Wallis
phase_angle_50khz_deg	1:0.5272; 2:0.1128; 3:0.1724; 4:NA	0.125233997	0.706260898	0.769404704	Kruskal-Wallis
phase_angle_slope_50_to_180	1:0.0405; 2:0.0321; 3:0.1174; 4:NA	0.806708686	0.716382681	0.806966357	Kruskal-Wallis
rs_mean_100_minus_50	1:0.1972; 2:0.008; 3:0.0034; 4:NA	0.406482775	0.440555573	0.060121948	Kruskal-Wallis
rs_mean_100_to_180_ratio	1:0.0001; 2:0.0; 3:0.0; 4:0.5951	0.188181044	0.30521902	0.229453225	Kruskal-Wallis
rs_mean_100khz	1:0.0; 2:0.0; 3:0.0011; 4:0.3245	0.26699403	0.312771527	0.425477636	Kruskal-Wallis
rs_mean_180_minus_100	1:0.0005; 2:0.0107; 3:0.1778; 4:0.0073	0.52800766	0.516696929	0.252610176	Kruskal-Wallis
rs_mean_180_minus_50	1:0.0411; 2:0.0053; 3:0.2075; 4:NA	0.420773697	0.47386574	0.240046976	Kruskal-Wallis
rs_mean_180khz	1:0.0; 2:0.0; 3:0.001; 4:0.2045	0.650439418	0.491024764	0.384290611	Kruskal-Wallis
rs_mean_50_to_100_ratio	1:0.0; 2:0.0; 3:0.0; 4:NA	0.418661604	0.388184051	0.024697101	Kruskal-Wallis
rs_mean_50_to_180_ratio	1:0.0; 2:0.0; 3:0.0; 4:NA	0.495058791	0.467718036	0.252293839	Kruskal-Wallis
rs_mean_50khz	1:0.0048; 2:0.0; 3:0.0029; 4:NA	0.396650339	0.584801659	0.706427333	Kruskal-Wallis
rs_mean_slope_100_to_180	1:0.0005; 2:0.0107; 3:0.1778; 4:0.0073	0.52800766	0.516696929	0.252610176	Kruskal-Wallis
rs_mean_slope_50_to_100	1:0.1972; 2:0.008; 3:0.0034; 4:NA	0.406482775	0.440555573	0.060121948	Kruskal-Wallis
rs_mean_slope_50_to_180	1:0.0411; 2:0.0053; 3:0.2075; 4:NA	0.420773697	0.47386574	0.240046976	Kruskal-Wallis
rs_sd_100khz	1:0.0; 2:0.0001; 3:0.0101; 4:0.5238	0.05418707	0.079969793	0.249518609	Kruskal-Wallis
rs_sd_180khz	1:0.0; 2:0.0; 3:0.0003; 4:0.9756	0.650025957	0.85680651	0.63534946	Kruskal-Wallis
rs_sd_50khz	1:0.0134; 2:0.0; 3:0.0138; 4:NA	0.343066122	0.563531336	0.786434588	Kruskal-Wallis
rs_sd_mean_across_freq	1:0.0; 2:0.0001; 3:0.2137; 4:0.1139	0.376686577	0.476618561	0.271364946	Kruskal-Wallis
xc_mean_100_minus_50	1:0.0203; 2:0.0027; 3:0.0004; 4:NA	0.756390016	0.335451851	0.15613678	Kruskal-Wallis
xc_mean_100_to_180_ratio	1:0.0; 2:0.0; 3:0.0; 4:0.8086	0.915142486	0.907382282	0.822429686	Kruskal-Wallis
xc_mean_100khz	1:0.0; 2:0.0; 3:0.0007; 4:0.6079	0.533410372	0.587654894	0.602411688	Kruskal-Wallis
xc_mean_180_minus_100	1:0.0; 2:0.0004; 3:0.0708; 4:0.5258	0.997310909	0.598533911	0.644498684	Kruskal-Wallis
xc_mean_180_minus_50	1:0.0629; 2:0.0019; 3:0.0477; 4:NA	0.999700046	0.527273658	0.522363005	Kruskal-Wallis
xc_mean_180khz	1:0.0; 2:0.0; 3:0.0073; 4:0.7142	0.594665142	0.533779726	0.186998643	Kruskal-Wallis
xc_mean_50_to_100_ratio	1:0.0; 2:0.0; 3:0.0; 4:NA	0.275279013	0.262177111	0.051717113	Kruskal-Wallis
xc_mean_50_to_180_ratio	1:0.0; 2:0.0; 3:0.0; 4:NA	0.157737838	0.147675373	0.393766584	Kruskal-Wallis
xc_mean_50khz	1:0.0; 2:0.0; 3:0.0008; 4:NA	0.781195641	0.844973689	0.858056106	Kruskal-Wallis
xc_mean_slope_100_to_180	1:0.0; 2:0.0004; 3:0.0708; 4:0.5258	0.997310909	0.598533911	0.644498684	Kruskal-Wallis
xc_mean_slope_50_to_100	1:0.0203; 2:0.0027; 3:0.0004; 4:NA	0.756390016	0.335451851	0.15613678	Kruskal-Wallis
xc_mean_slope_50_to_180	1:0.0629; 2:0.0019; 3:0.0477; 4:NA	0.999700046	0.527273658	0.522363005	Kruskal-Wallis

xc_sd_100khz	1:0.0; 2:0.0; 3:0.0163; 4:0.9815	0.884257732	0.922076716	0.647228396	Kruskal-Wallis
xc_sd_180khz	1:0.0; 2:0.0; 3:0.0004; 4:0.54	0.328023606	0.484153286	0.783402211	Kruskal-Wallis
xc_sd_50khz	1:0.0002; 2:0.0; 3:0.0031; 4:NA	0.747622001	0.76020189	0.784327254	Kruskal-Wallis
xc_sd_mean_across_freq	1:0.0; 2:0.0; 3:0.0055; 4:0.3738	0.110367299	0.456491141	0.658105079	Kruskal-Wallis
z_mag_100_minus_50	1:0.13; 2:0.0757; 3:0.002; 4:NA	0.5221956	0.367890755	0.099997899	Kruskal-Wallis
z_mag_100_to_180_ratio	1:0.0; 2:0.0; 3:0.0; 4:0.4444	0.172442605	0.335049815	0.303753554	Kruskal-Wallis
z_mag_100khz	1:0.0; 2:0.0; 3:0.0019; 4:0.754	0.373005626	0.408656969	0.407007019	Kruskal-Wallis
z_mag_180_minus_100	1:0.0001; 2:0.009; 3:0.2199; 4:0.2102	0.847417435	0.543040971	0.284316193	Kruskal-Wallis
z_mag_180_minus_50	1:0.2264; 2:0.0221; 3:0.2585; 4:NA	0.787131069	0.444146817	0.441492905	Kruskal-Wallis
z_mag_180khz	1:0.0; 2:0.0; 3:0.0116; 4:0.7528	0.616150618	0.4859713	0.212940886	Kruskal-Wallis
z_mag_50_to_100_ratio	1:0.0; 2:0.0; 3:0.0; 4:NA	0.210054364	0.181034867	0.021464362	Kruskal-Wallis
z_mag_50_to_180_ratio	1:0.0; 2:0.0; 3:0.0; 4:NA	0.261685511	0.243863701	0.320789881	Kruskal-Wallis
z_mag_50khz	1:0.0007; 2:0.0; 3:0.0068; 4:NA	0.650127627	0.807277077	0.80447277	Kruskal-Wallis
z_mag_slope_100_to_180	1:0.0001; 2:0.009; 3:0.2199; 4:0.2102	0.847417435	0.543040971	0.284316193	Kruskal-Wallis
z_mag_slope_50_to_100	1:0.13; 2:0.0757; 3:0.002; 4:NA	0.5221956	0.367890755	0.099997899	Kruskal-Wallis
z_mag_slope_50_to_180	1:0.2264; 2:0.0221; 3:0.2585; 4:NA	0.787131069	0.444146817	0.441492905	Kruskal-Wallis
pcv_pct	1:0.0; 2:0.0001; 3:0.0511; 4:0.4633	3.19E-05	8.38E-66	2.81E-31	Kruskal-Wallis
fecal_count_numeric	1:0.0; 2:0.0; 3:0.0008; 4:NA	0.001164522	0.000399211	0.000114229	Kruskal-Wallis

**Shapiro–Wilk and Levene tests were used as exploratory assumption diagnostics. Conventional ANOVA and Kruskal–Wallis p-values are presented for descriptive comparison only and should not be interpreted as confirmatory inferential results because repeated observations from the same animals violate the independence assumption of these tests. Selection of the primary inferential framework was based on the repeated-measures study design rather than preliminary assumption-test outcomes. Mixed-effects models incorporating animal identity as a random effect were used for the primary statistical analyses.*

Table S2. Spearman rank correlation coefficients (ρ) and associated p-values for the relationships between bioelectrical impedance analysis–derived variables and packed cell volume (PCV).

variable	*rho*	*p_value*
phase_angle_180khz_deg	-0.17065	0.023153
xc_mean_180khz	-0.15331	0.041627
rs_mean_slope_50_to_100	-0.19898	0.057237

rs_mean_100_minus_50	-0.19898	0.057237
z_mag_180khz	-0.14183	0.059694
rs_sd_100khz	-0.18632	0.064814
z_mag_100_minus_50	-0.19323	0.064964
z_mag_slope_50_to_100	-0.19323	0.064964
rs_mean_50_to_100_ratio	0.18791	0.072855
n_scans_100khz	-0.18055	0.073721
z_mag_50_to_100_ratio	0.187168	0.074013
rs_sd_mean_across_freq	-0.13249	0.075409
n_scans_180khz	-0.13112	0.081937
xc_mean_100_minus_50	-0.16524	0.115463
xc_mean_slope_50_to_100	-0.16524	0.115463
xc_mean_50_to_100_ratio	0.156443	0.136429
rs_mean_180khz	-0.10579	0.1611
xc_sd_100khz	-0.12591	0.214306
z_mag_100khz	-0.12419	0.220693
rs_mean_100khz	-0.12414	0.220856
n_scans_50khz	-0.11495	0.249965
xc_mean_100khz	-0.10726	0.290637
rs_sd_180khz	-0.06185	0.416164
xc_sd_mean_across_freq	-0.05908	0.429476
z_mag_50_to_180_ratio	0.069458	0.496758
rs_mean_50_to_180_ratio	0.067132	0.511316
phase_angle_100_to_180_ratio	-0.0652	0.523544
xc_mean_180_minus_100	0.064055	0.530909
xc_mean_slope_100_to_180	0.064055	0.530909
z_mag_slope_100_to_180	0.060368	0.554863
z_mag_180_minus_100	0.060368	0.554863
rs_mean_180_minus_100	0.059461	0.560837
rs_mean_slope_100_to_180	0.059461	0.560837
phase_angle_180_minus_100	0.053245	0.602573
xc_sd_50khz	0.049768	0.621137
xc_sd_180khz	-0.03728	0.62428
xc_mean_100_to_180_ratio	-0.04741	0.64294
xc_mean_50_to_180_ratio	0.043157	0.673061
xc_mean_50khz	0.034519	0.730522
phase_angle_100khz_deg	-0.02533	0.803468

phase_angle_50khz_deg	-0.02381	0.812254
xc_mean_180_minus_50	0.023815	0.815943
xc_mean_slope_50_to_180	0.023815	0.815943
phase_angle_50_to_180_ratio	-0.02314	0.821041
rs_mean_50khz	0.020578	0.837349
z_mag_50khz	0.016752	0.867278
phase_angle_50_to_100_ratio	0.017038	0.871932
phase_angle_slope_50_to_180	0.015508	0.879531
phase_angle_180_minus_50	0.015508	0.879531
z_mag_slope_50_to_180	-0.01452	0.887173
z_mag_180_minus_50	-0.01452	0.887173
phase_angle_100_minus_50	-0.00655	0.950591
rs_mean_100_to_180_ratio	-0.0062	0.951708
z_mag_100_to_180_ratio	-0.00542	0.957775
rs_sd_50khz	-0.00505	0.959987
rs_mean_180_minus_50	-0.00419	0.967374
rs_mean_slope_50_to_180	-0.00419	0.967374

**Spearman correlations were exploratory and did not directly account for repeated observations within animals. The reported p-values are unadjusted. After Benjamini–Hochberg correction across 57 comparisons, no association remained statistically significant (minimum FDR-adjusted p = 0.358). Animal-clustered bootstrap 95% confidence intervals for phase angle at 180 kHz and mean reactance at 180 kHz are reported in Section 3.5. These confidence intervals account for within-animal clustering but are not adjusted for multiple comparisons. The primary inferential interpretation is based on repeated-measures mixed-effects models.*

Table S3. *Classification performance across binary, three-class, and four-class FAMACHA© prediction tasks across model families and feature blocks.*

Model	*Balanced Accuracy*	*Macro F1*	*Weighted F1*	*ROC AUC*	*Average Precision*	*kappa*	*Feature block*	*Task*	*Quadratic weighted kappa*	*Macro Over_Auc*
Logistic	0.648	0.602	0.784	0.643	0.271	0.219	BIA_raw_only	binary		
SVM	0.513	0.513	0.772	0.661	0.238	0.026	BIA_raw_only	binary		
BPNN_MLP	0.505	0.505	0.778	0.598	0.225	0.011	BIA_raw_only	binary		
KNN	0.518	0.503	0.816	0.657	0.229	0.058	BIA_raw_only	binary		
RandomForest	0.508	0.494	0.806	0.745	0.281	0.024	BIA_raw_only	binary		
RandomForest	0.429	0.435	0.494				BIA_raw_only	multiclass_3class	0.272	0.653
BPNN_MLP	0.400	0.399	0.450				BIA_raw_only	multiclass_3class	0.244	0.546
SVM	0.378	0.374	0.444				BIA_raw_only	multiclass_3class	0.143	0.561
KNN	0.366	0.357	0.433				BIA_raw_only	multiclass_3class	0.118	0.582

RandomForest	0.305	0.306	0.464				BIA_raw_only	multiclass_4class	0.222	0.663
SVM	0.293	0.292	0.452				BIA_raw_only	multiclass_4class	0.103	0.451
KNN	0.281	0.276	0.438				BIA_raw_only	multiclass_4class	0.104	0.540
BPNN_MLP	0.236	0.237	0.365				BIA_raw_only	multiclass_4class	0.052	0.573
Logistic	0.565	0.522	0.720	0.550	0.165	0.084	BIA_raw_plus_derived	binary		
BPNN_MLP	0.512	0.512	0.785	0.577	0.196	0.025	BIA_raw_plus_derived	binary		
KNN	0.521	0.506	0.819	0.593	0.198	0.070	BIA_raw_plus_derived	binary		
SVM	0.505	0.505	0.778	0.499	0.138	0.011	BIA_raw_plus_derived	binary		
RandomForest	0.484	0.456	0.792	0.720	0.223	-0.048	BIA_raw_plus_derived	binary		
RandomForest	0.385	0.385	0.459				BIA_raw_plus_derived	multiclass_3class	0.113	0.638
SVM	0.334	0.328	0.397				BIA_raw_plus_derived	multiclass_3class	0.205	0.474
KNN	0.328	0.314	0.397				BIA_raw_plus_derived	multiclass_3class	0.133	0.545
BPNN_MLP	0.281	0.278	0.354				BIA_raw_plus_derived	multiclass_3class	0.017	0.465
RandomForest	0.300	0.302	0.472				BIA_raw_plus_derived	multiclass_4class	0.152	0.653
SVM	0.275	0.269	0.413				BIA_raw_plus_derived	multiclass_4class	0.164	0.394
KNN	0.251	0.241	0.401				BIA_raw_plus_derived	multiclass_4class	0.122	0.522
BPNN_MLP	0.222	0.219	0.366				BIA_raw_plus_derived	multiclass_4class	0.007	0.488
Logistic	0.623	0.567	0.752	0.657	0.208	0.164	BIA_plus_site	binary		
BPNN_MLP	0.527	0.530	0.803	0.613	0.188	0.067	BIA_plus_site	binary		
SVM	0.508	0.508	0.782	0.514	0.145	0.018	BIA_plus_site	binary		
KNN	0.521	0.506	0.819	0.597	0.199	0.070	BIA_plus_site	binary		
RandomForest	0.511	0.497	0.809	0.765	0.284	0.035	BIA_plus_site	binary		
RandomForest	0.393	0.392	0.469				BIA_plus_site	multiclass_3class	0.178	0.652
SVM	0.325	0.320	0.385				BIA_plus_site	multiclass_3class	0.187	0.491
BPNN_MLP	0.319	0.319	0.368				BIA_plus_site	multiclass_3class	0.055	0.482
KNN	0.329	0.311	0.395				BIA_plus_site	multiclass_3class	0.135	0.526
RandomForest	0.302	0.303	0.476				BIA_plus_site	multiclass_4class	0.193	0.667
SVM	0.268	0.263	0.402				BIA_plus_site	multiclass_4class	0.176	0.402
KNN	0.252	0.239	0.399				BIA_plus_site	multiclass_4class	0.125	0.505
BPNN_MLP	0.213	0.213	0.330				BIA_plus_site	multiclass_4class	0.053	0.492
Logistic	0.698	0.622	0.784	0.783	0.324	0.268	BIA_plus_site_plus_weather	binary		
BPNN_MLP	0.531	0.533	0.806	0.625	0.189	0.076	BIA_plus_site_plus_weather	binary		
RandomForest	0.529	0.528	0.817	0.800	0.281	0.085	BIA_plus_site_plus_weather	binary		
SVM	0.496	0.491	0.735	0.583	0.152	-0.007	BIA_plus_site_plus_weather	binary		
KNN	0.497	0.463	0.803	0.600	0.154	-0.011	BIA_plus_site_plus_weather	binary		
RandomForest	0.375	0.374	0.448				BIA_plus_site_plus_weather	multiclass_3class	0.196	0.662
BPNN_MLP	0.339	0.338	0.392				BIA_plus_site_plus_weather	multiclass_3class	0.054	0.549
SVM	0.340	0.334	0.400				BIA_plus_site_plus_weather	multiclass_3class	0.299	0.560
KNN	0.336	0.315	0.403				BIA_plus_site_plus_weather	multiclass_3class	0.125	0.530
RandomForest	0.296	0.298	0.464				BIA_plus_site_plus_weather	multiclass_4class	0.224	0.668

SVM	0.284	0.272	0.424				BIA_plus_site_plus_weather	multiclass_4class	0.256	0.480
BPNN_MLP	0.241	0.239	0.387				BIA_plus_site_plus_weather	multiclass_4class	0.010	0.590
KNN	0.249	0.227	0.403				BIA_plus_site_plus_weather	multiclass_4class	0.100	0.508
Logistic	0.702	0.648	0.812	0.791	0.472	0.307	BIA_plus_site_plus_other	binary		
BPNN_MLP	0.596	0.612	0.836	0.716	0.292	0.229	BIA_plus_site_plus_other	binary		
SVM	0.566	0.574	0.816	0.732	0.271	0.150	BIA_plus_site_plus_other	binary		
KNN	0.521	0.506	0.819	0.665	0.243	0.070	BIA_plus_site_plus_other	binary		
RandomForest	0.511	0.497	0.809	0.807	0.333	0.035	BIA_plus_site_plus_other	binary		
SVM	0.384	0.385	0.437				BIA_plus_site_plus_other	multiclass_3class	0.268	0.555
RandomForest	0.365	0.354	0.450				BIA_plus_site_plus_other	multiclass_3class	0.199	0.672
BPNN_MLP	0.346	0.348	0.392				BIA_plus_site_plus_other	multiclass_3class	0.239	0.525
KNN	0.355	0.336	0.426				BIA_plus_site_plus_other	multiclass_3class	0.183	0.565
RandomForest	0.316	0.321	0.495				BIA_plus_site_plus_other	multiclass_4class	0.225	0.682
SVM	0.300	0.298	0.443				BIA_plus_site_plus_other	multiclass_4class	0.270	0.461
BPNN_MLP	0.279	0.280	0.412				BIA_plus_site_plus_other	multiclass_4class	0.208	0.624
KNN	0.271	0.258	0.432				BIA_plus_site_plus_other	multiclass_4class	0.178	0.542
Logistic	0.707	0.677	0.840	0.841	0.529	0.357	Full_without_PCV	binary		
SVM	0.552	0.547	0.782	0.690	0.200	0.095	Full_without_PCV	binary		
BPNN_MLP	0.540	0.546	0.817	0.756	0.344	0.107	Full_without_PCV	binary		
RandomForest	0.535	0.536	0.824	0.838	0.366	0.109	Full_without_PCV	binary		
KNN	0.500	0.464	0.806	0.631	0.201	0.000	Full_without_PCV	binary		
RandomForest	0.394	0.397	0.460				Full_without_PCV	multiclass_3class	0.246	0.681
BPNN_MLP	0.400	0.393	0.400				Full_without_PCV	multiclass_3class	0.230	0.531
SVM	0.363	0.361	0.422				Full_without_PCV	multiclass_3class	0.339	0.563
KNN	0.346	0.326	0.413				Full_without_PCV	multiclass_3class	0.156	0.547
SVM	0.312	0.310	0.457				Full_without_PCV	multiclass_4class	0.305	0.504
BPNN_MLP	0.302	0.308	0.424				Full_without_PCV	multiclass_4class	0.260	0.598
RandomForest	0.300	0.304	0.470				Full_without_PCV	multiclass_4class	0.279	0.683
KNN	0.256	0.232	0.412				Full_without_PCV	multiclass_4class	0.129	0.528

**Overall, multiclass performance was modest across all models, with macro F1 values generally ranging from approximately 0.31 to 0.44. Random Forest produced the strongest performance in most feature blocks, including BIA_raw_only (macro F1 = 0.435), BIA_raw_plus_derived (0.385), BIA_plus_site (0.392), and BIA_plus_site_plus_weather (0.374), while SVM achieved the highest macro F1 in BIA_plus_site_plus_other (0.385). In the Full_without_PCV feature block, Random Forest and BPNN-MLP showed comparable performance, with macro F1 values of 0.397 and 0.393, respectively. These results indicate that adding site, weather, and other non-PCV contextual variables produced only limited gains in multiclass discrimination, and that three-class FAMACHA prediction remained substantially more challenging than binary classification in this dataset.*

Table S4. Permutation-based variable importance rankings for the best-performing binary FAMACHA classification model. Importance values represent the mean decrease in model performance after random permutation of each feature, and the accompanying standard deviation reflects variability across repeated permutations.

Feature	importance means	importance_sd
Fecal_Count_Numeric	0.134	0.016
Avg_Rainfall_In	0.119	0.033
Avg_Wind_Speed_Mph	0.095	0.025
Xc_Sd_100khz	0.063	0.018
Avg_Relative_Humidity_Pct	0.037	0.018
Phase_Angle_50khz_Deg	0.036	0.019
Phase_Angle_50_To_100_Ratio	0.031	0.019
Rs_Sd_180khz	0.026	0.017
Xc_Mean_50_To_100_Ratio	0.025	0.003
N_Scans_180khz	0.022	0.021

Rs_Mean_100_To_180_Ratio	0.021	0.015
Avg_Dew_Point_C	0.019	0.015
High_Temp_C	0.007	0.003
Phase_Angle_50_To_180_Ratio	0.006	0.004
Xc_Mean_50_To_180_Ratio	0.006	0.003
Rs_Sd_50khz	0.006	0.004
Phase_Angle_180_Minus_50	0.006	0.006
Phase_Angle_Slope_50_To_180	0.006	0.006
Xc_Mean_100_To_180_Ratio	0.006	0.018
N_Scans_50khz	0.005	0.014
Rs_Mean_50_To_180_Ratio	0.005	0.008
Phase_Angle_100_Minus_50	0.005	0.002
Xc_Mean_180_Minus_50	0.005	0.004
Xc_Mean_Slope_50_To_180	0.005	0.004
Phase_Angle_100khz_Deg	0.004	0.005
Z_Mag_180_Minus_50	0.004	0.003
Z_Mag_Slope_50_To_180	0.004	0.003
Phase_Angle_100_To_180_Ratio	0.004	0.007
Rs_Mean_Slope_100_To_180	0.004	0.008
Rs_Mean_180_Minus_100	0.004	0.008
Xc_Mean_180khz	0.003	0.013
Xc_Mean_50khz	0.003	0.000
Low_Temp_C	0.003	0.003
Phase_Angle_180_Minus_100	0.003	0.008
Xc_Mean_100khz	0.003	0.010
Rs_Mean_50_To_100_Ratio	0.003	0.007
Rs_Mean_180_Minus_50	0.003	0.005
Rs_Mean_Slope_50_To_180	0.003	0.005
Xc_Sd_Mean_Across_Freq	0.002	0.001
Avg_Wind_Direction_Sin	0.002	0.002
Avg_Wind_Direction_Cos	0.002	0.002
Avg_Wind_Direction_Angle_Deg	0.002	0.002
N_Scans_100khz	0.001	0.003

Rs_Sd_Mean_Across_Freq	0.001	0.007
Z_Mag_Slope_100_To_180	0.001	0.008
Z_Mag_180_Minus_100	0.001	0.008
Z_Mag_100_To_180_Ratio	0.001	0.009
Xc_Mean_Slope_50_To_100	0.001	0.002
Xc_Mean_100_Minus_50	0.001	0.002
Rs_Mean_Slope_50_To_100	0.001	0.001
Rs_Sd_100khz	0.001	0.001
Rs_Mean_100_Minus_50	0.001	0.001
Z_Mag_Slope_50_To_100	0.001	0.002
Z_Mag_100_Minus_50	0.001	0.002
Z_Mag_50_To_100_Ratio	0.001	0.001
Z_Mag_50_To_180_Ratio	0.000	0.002
Xc_Sd_180khz	0.000	0.003
Avg_Soil_Moisture_12in_Pct	0.000	0.000
Weather_Condition	0.000	0.000
Avg_Temp_C	0.000	0.000
Rs_Mean_180khz	-0.001	0.009
Z_Mag_180khz	-0.002	0.010
Phase_Angle_180khz_Deg	-0.002	0.010
Avg_Total_Radiation_Mj_M2	-0.004	0.003
Z_Mag_50khz	-0.008	0.014
Rs_Mean_100khz	-0.008	0.013
Z_Mag_100khz	-0.009	0.011
Xc_Mean_Slope_100_To_180	-0.010	0.010
Xc_Mean_180_Minus_100	-0.010	0.010
Site_Analysis	-0.010	0.005
Rs_Mean_50khz	-0.011	0.011
Avg_Soil_Temp_2in_C	-0.012	0.008
Avg_Soil_Temp_4in_C	-0.014	0.009
Xc_Sd_50khz	-0.014	0.009

Supplementary Table S5. *Ablation performance of feature blocks for binary objective FAMACHA© risk classification.*

Feature_block	n_features	ROC_AUC	AUC_CI_low	AUC_CI_high	Balanced_accuracy	Precision	Recall_sensitivity	F1	Brier_score	TN	FP	FN	TP
BIA only	42	0.67	0.55	0.78	0.58	0.20	0.42	0.27	0.21	116	41	14	10
BIA + FEC	43	0.76	0.64	0.86	0.68	0.32	0.54	0.40	0.16	129	28	11	13
BIA + Weather/THI	58	0.78	0.67	0.86	0.66	0.27	0.54	0.36	0.17	122	35	11	13
BIA + Raster	47	0.66	0.54	0.76	0.59	0.21	0.42	0.28	0.21	119	38	14	10
FEC + Weather/THI	17	0.84	0.73	0.92	0.73	0.28	0.75	0.41	0.15	111	46	6	18
Full multimodal	64	0.82	0.71	0.90	0.69	0.34	0.54	0.42	0.13	132	25	11	13

Supplementary Table S6. *Threshold-based decision-support performance of the full multimodal model for binary objective FAMACHA© risk classification.*

Threshold_rule	threshold	Sensitivity	Specificity	Balanced_accuracy	Precision	F1	TN	FP	FN	TP
Youden J	0.13	0.83	0.71	0.77	0.31	0.45	112	45	4	20
Sensitivity >=0.80, max specificity	0.13	0.83	0.71	0.77	0.31	0.45	112	45	4	20

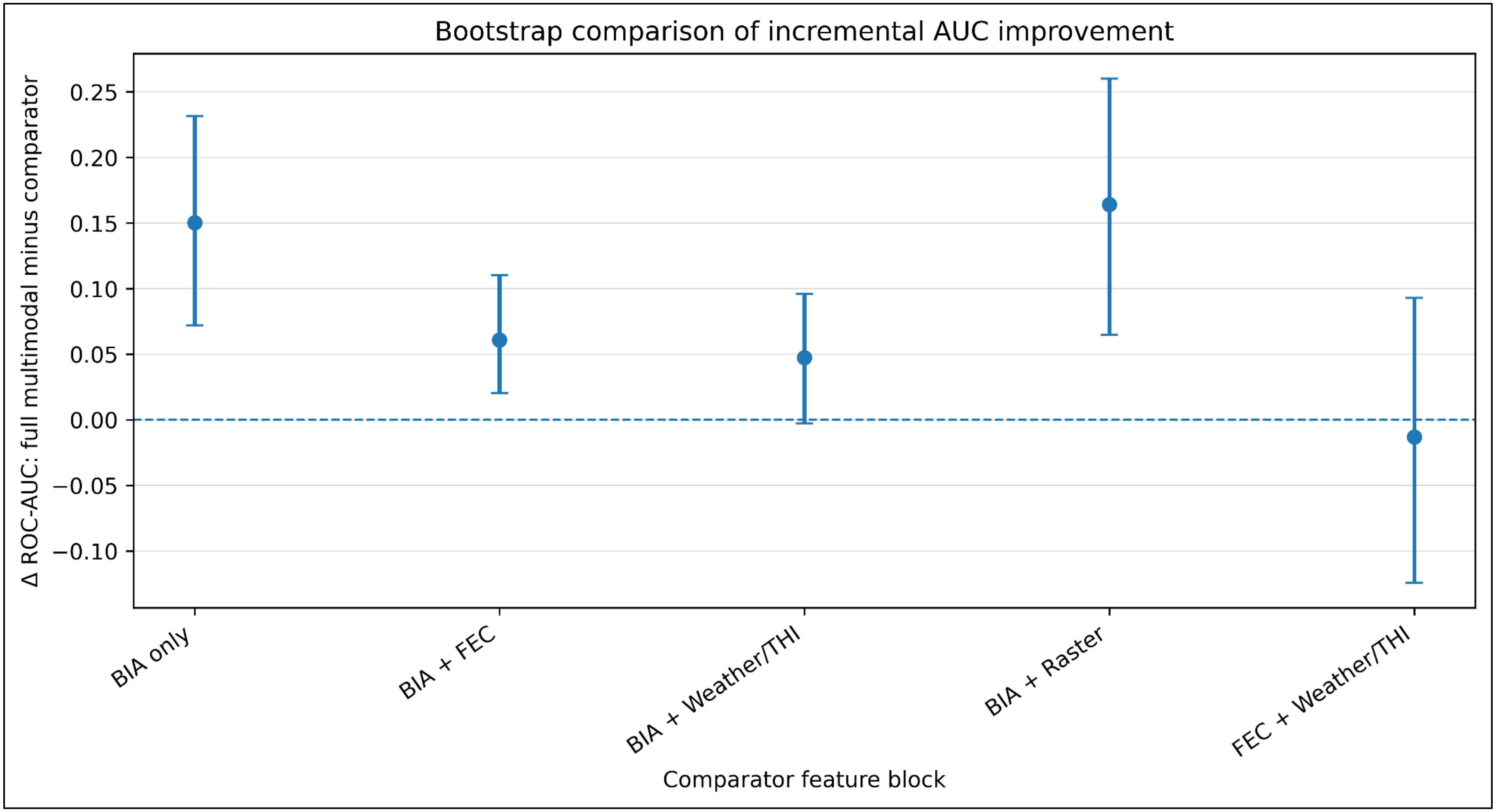

Supplementary Figure S1. Bootstrap comparison of incremental ROC-AUC improvement for the full multimodal model relative to reduced feature blocks. Points show mean ΔAUC values, and error bars show bootstrap 95% confidence intervals. The full multimodal model significantly improved over BIA-only, BIA plus fecal count, and BIA plus raster-derived feature blocks, but did not significantly outperform the fecal count plus weather/THI feature block.

Supplementary Table S7. Matched conventional versus BIA-augmented feature-block comparisons for binary objective FAMACHA anemia-risk classification.

Comparison	n (obs.)	n (goats)	Conventional ROC-AUC	With BIA ROC-AUC	ΔAUC	95% CI for ΔAUC
All labeled; all BIA features	181	79	0.853	0.870	+0.016	-0.052 to +0.082
All labeled; BIA without scan counts	181	79	0.853	0.855	+0.001	-0.073 to +0.069
Measured-FEC subset; all BIA features	76	58	0.723	0.770	+0.048	-0.107 to +0.213
Measured-FEC subset; BIA without scan counts	76	58	0.723	0.786	+0.064	-0.074 to +0.218

Note: ΔAUC = ROC-AUC (conventional features + BIA) − ROC-AUC (conventional features). Confidence intervals were obtained by paired animal-clustered bootstrap resampling. All 95% confidence intervals include zero; none establishes a reliable incremental discrimination benefit. The primary comparisons include within-fold imputation of missing FEC values, whereas the measured-FEC comparisons refit models on the complete-FEC subset (76 observations from 58 goats; 22 high-risk).

Supplementary Table S8. Sensitivity of the matched incremental BIA effect to 20 alternative animal-grouped cross-validation partitions (full 181-observation cohort).

Group-split random seed	Conventional ROC-AUC	With BIA ROC-AUC	ΔAUC
**2026**	0.831	0.767	-0.064
**2027**	0.844	0.873	+0.029
**2028**	0.848	0.783	-0.064
**2029**	0.861	0.889	+0.029
**2030**	0.863	0.864	+0.000
**2031**	0.869	0.868	-0.001
**2032**	0.852	0.865	+0.012
**2033**	0.840	0.825	-0.014
**2034**	0.833	0.843	+0.010
**2035**	0.848	0.865	+0.017
**2036**	0.842	0.869	+0.027
**2037**	0.835	0.816	-0.019
**2038**	0.856	0.876	+0.020
**2039**	0.842	0.825	-0.016
**2040**	0.869	0.729	-0.140
**2041**	0.865	0.814	-0.051
**2042**	0.838	0.845	+0.007

**2043**	0.857	0.845	-0.012
**2044**	0.856	0.840	-0.016
**2045**	0.835	0.870	+0.035

Across the 20 alternative partitions, mean ΔAUC was -0.011 (median -0.0002), with a range from -0.140 to +0.035; ROC-AUC improved with BIA in 10 of 20 partitions. These alternative split results describe sensitivity to data partitioning and must not be interpreted as independent validation cohorts or as a bootstrap confidence interval.

Supplementary Table S9. Animal-level representation, repeated-observation structure, and variation in objective FAMACHA anemia-risk categories.

Measure	Count	Interpretation
**Analysis-ready observations**	183	81 goats; includes 2 unlabeled observations
**Labeled observations for classification**	181	79 goats; 157 low-risk and 24 high-risk observations
**Class 1**	85 observations; 40 goats	Goats may contribute to more than one category
**Class 2**	72 observations; 41 goats	Goats may contribute to more than one category
**Class 3**	21 observations; 19 goats	High-risk classification
**Class 4**	3 observations; 3 goats	Severe category; very limited representation
**Low-risk (classes 1–2)**	157 observations; 69 goats	Includes 11 goats later/earlier classified as high risk
**High-risk (classes 3–4)**	24 observations; 21 goats	Includes 11 goats later/earlier classified as low risk
**Goats contributing 1 labeled observation**	33 goats	Among 79 goats with complete outcome labels
**Goats contributing 2 labeled observations**	15 goats	Counts reflect animal-by-date-by-site rows
**Goats contributing 3 labeled observations**	13 goats	Counts reflect animal-by-date-by-site rows
**Goats contributing 4 labeled observations**	11 goats	Counts reflect animal-by-date-by-site rows
**Goats contributing 5 labeled observations**	7 goats	Counts reflect animal-by-date-by-site rows
**Goats with data from 1 sampling date**	48 goats	Includes goats with multiple anatomical sites on the date
**Goats with data from 2 sampling dates**	23 goats	Independent sampling occasions
**Goats with data from 3 sampling dates**	8 goats	Independent sampling occasions
**Goats crossing low- and high-risk states across dates**	11 of 31 longitudinally observed goats	35.5% of goats sampled on ≥2 dates; risk status changed over time
**Animal-by-date combinations**	118	Distinct animal-date sampling occasions across the 181 labeled records

Note: Animal counts across the four FAMACHA categories and the two risk groups are not additive because some goats contributed observations in multiple categories. The distribution of labeled observations per goat was 1–5 (median 2; mean 2.29). The 11 risk-state changes were assessed across distinct collection dates, not inferred from measurements at multiple anatomical sites within one date.

Supplementary Table S10. Observed risk-class composition of the reconstructed five-fold animal-grouped cross-validation test partitions (n = 181 labeled observations from 79 goats).

Fold	Test obs.	Test goats	Low-risk obs.	High-risk obs.	High-risk goats	Class 1 obs.	Class 2 obs.	Class 3 obs.	Class 4 obs.
1	37	16	31	6	5	19	12	5	1
2	36	15	32	4	3	16	16	3	1

3	36	16	30	6	5	10	20	6	0
4	36	16	33	3	3	24	9	3	0
5	36	16	31	5	5	16	15	4	1
